# Novel quinazolin-4-one based derivatives bearing 1,2,3-triazole and glycoside moieties as potential cytotoxic agents through dual EGFR and VEGFR-2 inhibitory activity

**DOI:** 10.1038/s41598-024-73171-8

**Published:** 2024-10-23

**Authors:** Adel A.-H. Abdel-Rahman, Mohamed N. El-Bayaa, Asmaa Sobhy, Eman M. El-Ganzoury, Eman S. Nossier, Hanem M. Awad, Wael A. El-Sayed

**Affiliations:** 1 Chemistry Department, Faculty of Science, Menoufia University, Shebin El-Kom, Egypt; 2 Department of Chemistry, College of Science, Qassim University, Buraidah, 51452 Saudi Arabia; 3 Department of Pharmaceutical Medicinal Chemistry and Drug Design, Faculty of Pharmacy (Girls), Al-Azhar University, Cairo, 11754 Egypt; 4 The National Committee of Drugs, Academy of Scientific Research and Technology, Cairo, 11516 Egypt; 5 Tanning Materials and Leather Technology Department, National Research Centre, Dokki, Giza, 12622 Egypt

**Keywords:** Quinazolin-4-one, 1,2,3-Triazole, Glycosides, Cytotoxicity, EGFR, VEGFR-2, Medicinal chemistry, Organic chemistry

## Abstract

The toxicity that was caused by the developed medications for anticancer treatment is, unfortunately, an earnest problem stemming from the various involved targets, and accordingly, intense research for overcoming such a phenomenon remains indispensable. In the current inquiry, an innovative category of substituted quinazoline-based glycosides incorporating a core of 1,2,3-triazole and attached to distinct acetylated likewise deprotected sugar segments are created and produced synthetically. The resulted 1,2,3-triazolyl-glycosides products were investigated for their ability to cause cytotoxicity to several human cancer cell lines. The quinazoline based glycosyl-1,2,3-triazoles **10**–**13** with free hydroxy sugar moiety revealed excellent potency against (IC_50 range_ = 5.70–8.10 µM, IC_50 Doxorubicin_ = 5.6 ± 0.30 µM, IC_50 Erlotinib_ = 4.3 ± 0.1 µM). against MCF-7 cancer cell line. In addition, the derived glycosides incorporating quinazolinone and triazole core **6**–**13** with acetylated and deprotected sugar parts showed excellent and superior potency against HCT-116 (IC_50 range_ = 2.90–6.40 µM). The potent products were revealed as safe cytotoxic agents as indicated by their studied safety profiles. Additional research of promising candidates inhibitory analysis performed against EGFR and VEGFR-2. The hydroxylated glycosides incorporating triazole and quinazoline system **11** and **13** with *N*-methyl substitution of quinazolinone, gave excellent potency against EGFR (IC_50_ = 0.35 ± 0.11 and 0.31 ± 0.06 µM, correspondingly) since glycoside 13 revealed comparable IC_50_ (3.20 ± 0.15 µM) to sorafenib against VEGFR-2. For more understanding of its action mode, it was analyzed how the 1,2,3-triazolyl glycoside **13** made an effect on the apoptosis induction and the arrest of the cell cycle. It was revealed that it had the ability to stop HCT-116 cells in their cell cycle’s G1 stage. Moreover, the influence of quinazolinone-1,2,3-triazole-glycoside **13** upon p53, Bax, and Bcl-2 levels in HCT-116 units was also studied for future approaches toward its behavior. Additionally, the latter derivative may trigger apoptosis, as indicated by a significant increase in apoptotic cells. Furthermore, molecular docking was simulated to make an obvious validation and comprehension acquirement of the binding’s characteristics also attractions among the most forceful compounds side by side with their aimed enzymes.

**Supplementary Information:**

The online version contains supplementary material available at 10.1038/s41598-024-73171-8.

## Introduction

Cancer is seriously the most dangerous sickness spreading everywhere on the globe, obviously being the second leading cause of human death worldwide. Cancer is characterized by aberrant division of cells and death that results in abnormal unit proliferation^1^. There are several therapeutic methods for treating cancer including, chemotherapy, surgery, radiotherapy, and immunotherapy, however, effective chemotherapy medications with minimal side effects occupy the majority of attention^2^. Trying to reach the most potent agents possessing no or minimum side effects. By understanding the characteristics of the molecular and cellular causes of cancer, the development of cancer remedies becomes more advanced and effective. The improvement and progress of different kinds of cancer are directed by the aberrant activation of a number of key regulatory signaling proteins such as receptor tyrosine kinases (RTKs), platelet-derived growth factor receptors (PDGFRs), vascular endothelial growth factor receptor (VEGFR), and epidermal growth factor receptor (EGFR). The latter fact represents a beneficial mechanism that renders these receptors attractive candidates for treatment^3^.

Epidermal growth factor receptor (EGFR), also known as ErbB1 or HER1, is a transmembrane receptor tyrosine kinase from the ErbB family^4^. ErbB receptors’ extracellular domains are bound to certain ligands or growth factors that stimulate dimerization and autophosphorylation. This process activates the cytoplasmic tyrosine kinase domains and initiates downstream signalling pathways that control cell growth, differentiation, migration, and apoptosis^5,6^. Since EGFR-mediated signal transduction stimulates tumour cell proliferation, local invasion, angiogenesis, and metastasis, it has been linked to a number of human malignancies. A poor outcome is typically attributed to EGFR overexpression and/or mutation, which are clinical characteristics of many solid tumors^7^. Additionally, vascular endothelial growth factor (VEGF), the main inducer of functional angiogenesis, is stimulated by EGFR signalling pathways^8^.

In solid tumors, VEGF induces endothelial cell activation, migration, and proliferation as well as raising vascular permeability. The critical stage in this process is facilitated by a particular receptor called VEGFR-2 (vascular endothelial growth factor receptor 2)^9^. EGFR and VEGFR-2 appear closely related, having similar downstream signal transduction pathways and being crucial regulators in angiogenesis and tumor formation^7^. The antitumoral action of EGFR inhibitors is partially attributed to the suppression of the VEGFR-2 signalling pathway, while one potential resistance mechanism to anti-EGFR therapy is the independent stimulation of VEGF expression^10,11^. Therefore, EGFR and VEGFR-2 kinases are recognised targets in cancer therapy, with many FDA-approved inhibitors for clinical usage in solid tumors overexpressing EGFR and/or VEGFR-2^12,13^.

**Fig. 1:**
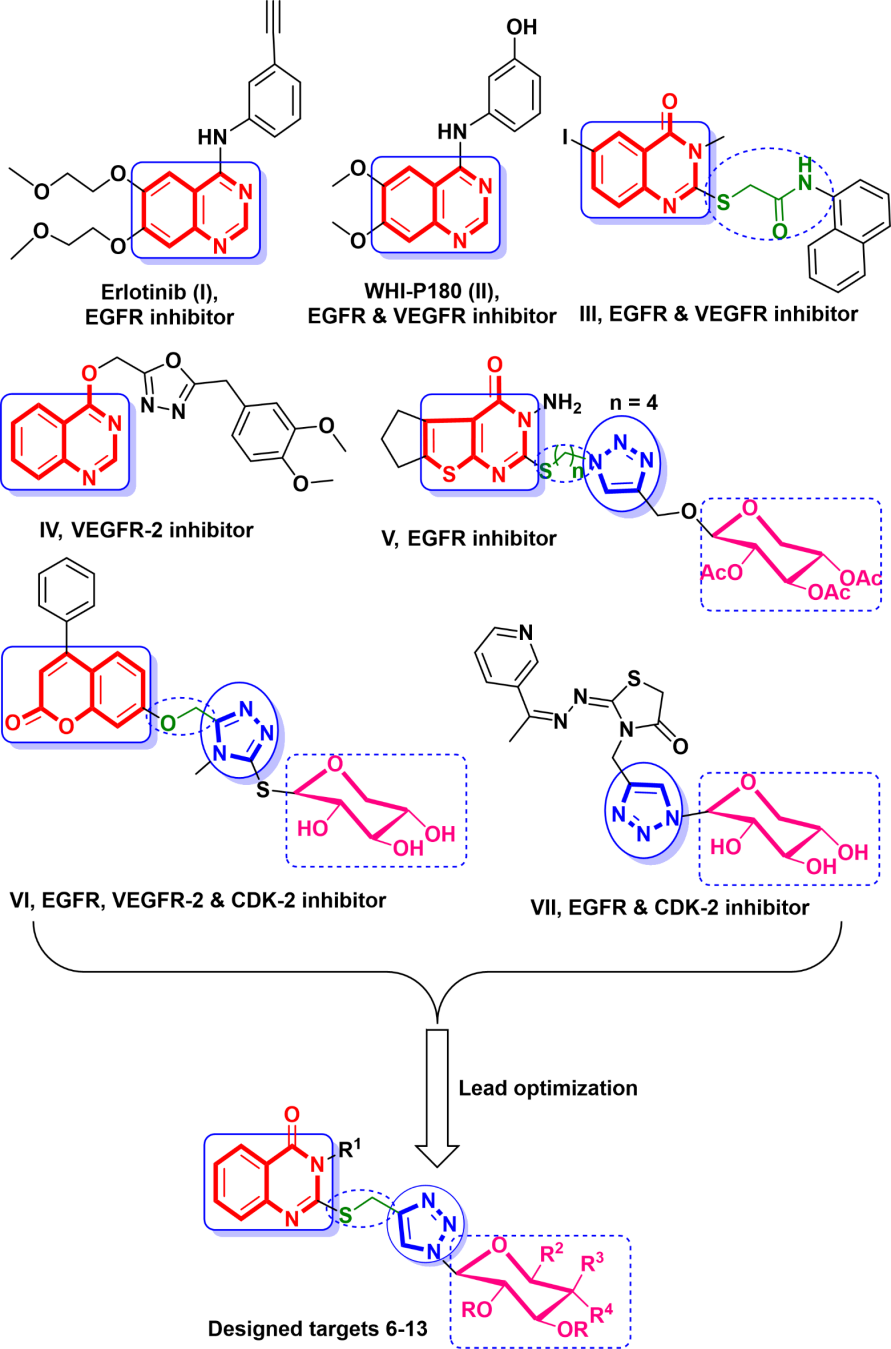
Reported quinazoline, triazole and/or glycoside based compounds and designed quinazolinone-1,2,3-triazole-glycosides as EGFR and VEGFR-2 inhibitors.

Quinazolines attract great attention because of their selectivity and potency in chemotherapy^14,15^. Erlotinib **I**, lapatinib, and gefitinib are FDA-approved cancer-related medicines containing a quinazoline core that inhibit EGFR^16–23^. In addition, quinazoline-based compounds such as WHI-P180 (**II**) and **III** have been characterized with their potent anticancer activity through inhibition of both EGFR and VEGFR-2 kinases^24,25^. Quinazoline **IV** revealed excellent cellular activity with an IC_50_ value of 0.23 µM against MCF-7 cell line superior to the positive control tivozanib through its inhibitory activity against VEGFR-2^26,27^. Other heterocyclic-based compounds including 1,2,3-triazole^28–38^ and glycoside motifs^39–43^ were found as effective building blocks in many bioactivities especially anticancer one. A number of reported structures incorporating 1,2,3-triazole and sugar moieties as nucleoside analogues **V**-**VII** displayed potent cytotoxicity against different human cancer cell lines through their inhibitory efficiency upon EGFR, VEGFR-2 and/or CDK-2 kinases^44–46^ (Fig. 1).

Based upon the structural features of the leads **V** and **VI**, ring variation of their correspondence cyclopenta^4,5^ thieno[2,3-*d*]pyrimidinone and coumarin with the significant quinazolinone and keeping 1,2,3-triazole and glycoside moities, a new series of quinazolinone-1,2,3-triazole glycoside hybrids with thiomethyl linker were designed and chemically synthesized. Their antiproliferative activities were evaluated against a variety of human cancerous and normal cell lines. The promising derivatives were further subjected to assessment against EGFR and VEGFR-2 as well as apoptotic indicators Bax, Bcl-2 and p53 to concise their mechanism of action. Additional in silico studies were supplied to get more information about optimum binding interactions with the screened enzymes and drug likness.

## Results and discussion

### Chemistry

The hybridization approach of molecules was applied as a useful instrument to afford the targeted products incorporating the quinazoline, 1,2,3-triazole and glycopyranosyl systems in one structure. The 1,3-dipolar cycloaddition strategy was employed to create the 1,2,3-triazole core structure through click-reaction in the targeted molecules. The required functionalities; terminal acetylenic and azido centers necessary for the click reaction were achieved in the terminal acetylenes **3**, **4** and the glycosyl azides **5a**,** b**. Thus, the crucial acetylenic terminal active center was produced through the interaction of propargyl bromide with quinazoline-thiols **1** and **2** in an alkaline medium.

By using Cu(I) to catalyze 1,3-dipolar cycloaddition methodologies according to click-chemistry conditions^47^, the glycopyranosyl azides, including xylopyranosyl azides or 2,3,4,6-tetra-*O*‐acetyl‐*D*-galacto, reacted with the *S*-alkyne compounds **3** and **4** to produce the desired 1,2,3-triazole-*N*-glycoides based quinazoline structure. In the later step, a technique utilizing copper sulfate pentahydrate and sodium ascorbate were employed to generate the necessary catalytic Cu(I) species by reducing copper (II) salt in the process for catalyzing the click reaction. The optimum solvent that yielded the most amounts of the desired click-products was Tetrahydrofuran /water (2:1) blended solvent **6**–**9** with the known regioselectivity of the creation of the click yields. The *N*1-glycosyl 1,2,3 triazoles linked to the substituted quinazoline system’s ^1^H-NMR spectra revealed indications for the glycopyranosyl hydrogens, acetylenic-methyl protons, and other hydrogens in the quinazoline part.

The values assigned for the anomeric proton were detected between 6.16 and 6.22 ppm while the anomeric carbon values were revealed at 84.17–84.66. The coupling constants (*J* values) for the glycopyranosyl ring’s anomeric hydrogen (H-1) for the glycosides were detected between 9.5 and 10.2 Hz which also revealed the nature of the *β*‐*N*‐glycosidic attachment between the triazole ring and the glycopyranosyl ring.

**Scheme 1:**
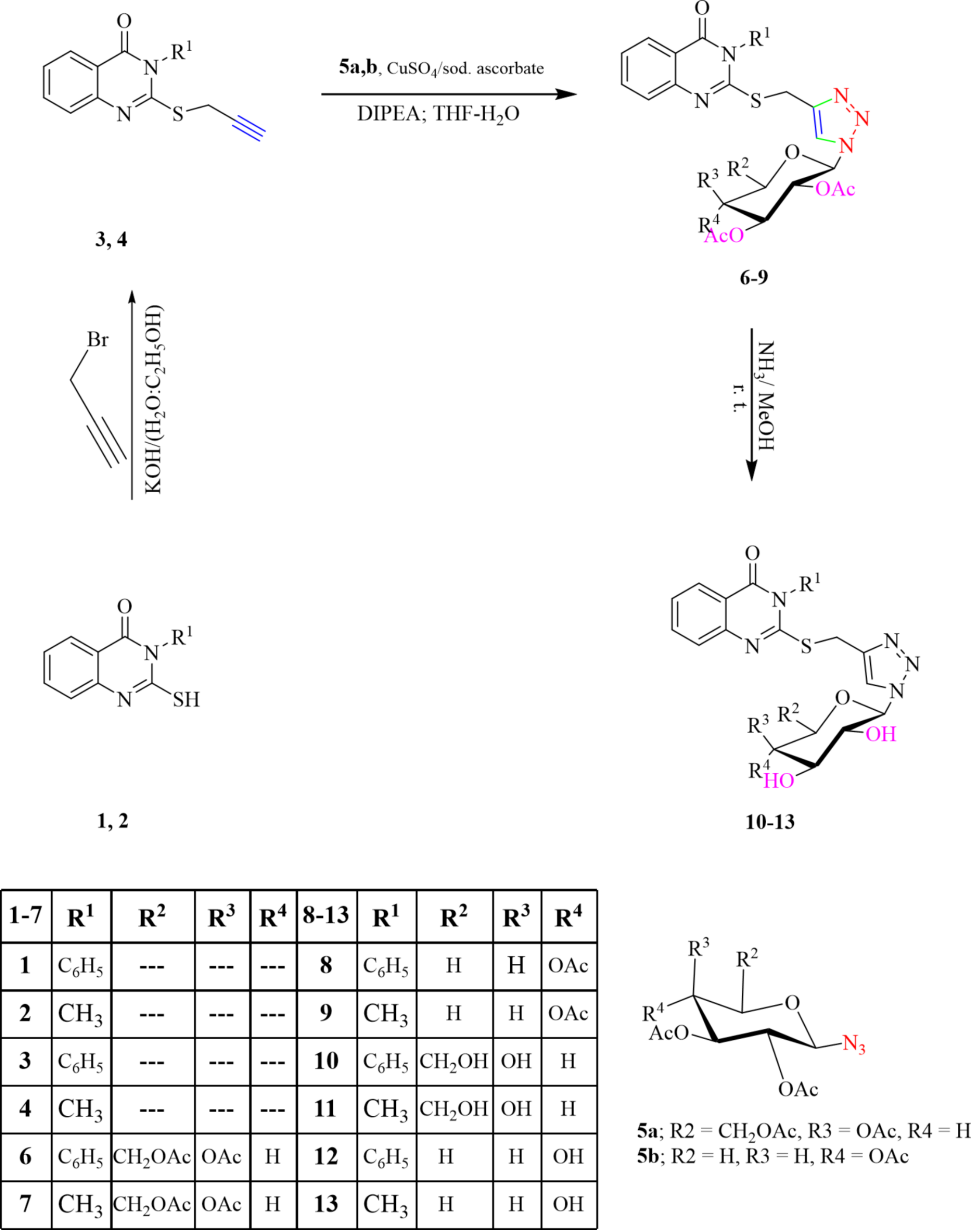
Synthesis of quinazolin-4-ones schlepping on 1,2,3-triazole and/or glycoside components.

Deacetylation of 1,2,3-triazole glycosides were synthesized at room temperature in presence of saturated ammonia/methanol mixture **6**–**9**, yielding the corresponding 1,2,3-triazole‐*N*‐glycosides **10**–**13** with free hydroxylated sugar moieties (Scheme 1). The deacetylation process was verified by the lack of the CH_3_C = O groups’ carbonyl band, in the sugar moiety, in their designated infrared spectra and instead the existence of distinct absorption frequencies related to free hydroxyl groups. The ^1^H-NMR spectra displayed The electromagnetic signals ascribed to the hydroxyl protons and signified the acetyl groups disappearing in their safeguarding precursors, matching the free hydroxyl glycosides’ structures that were produced.

### Anticancer activity

#### Cytotoxic activity

**Fig. 2:**
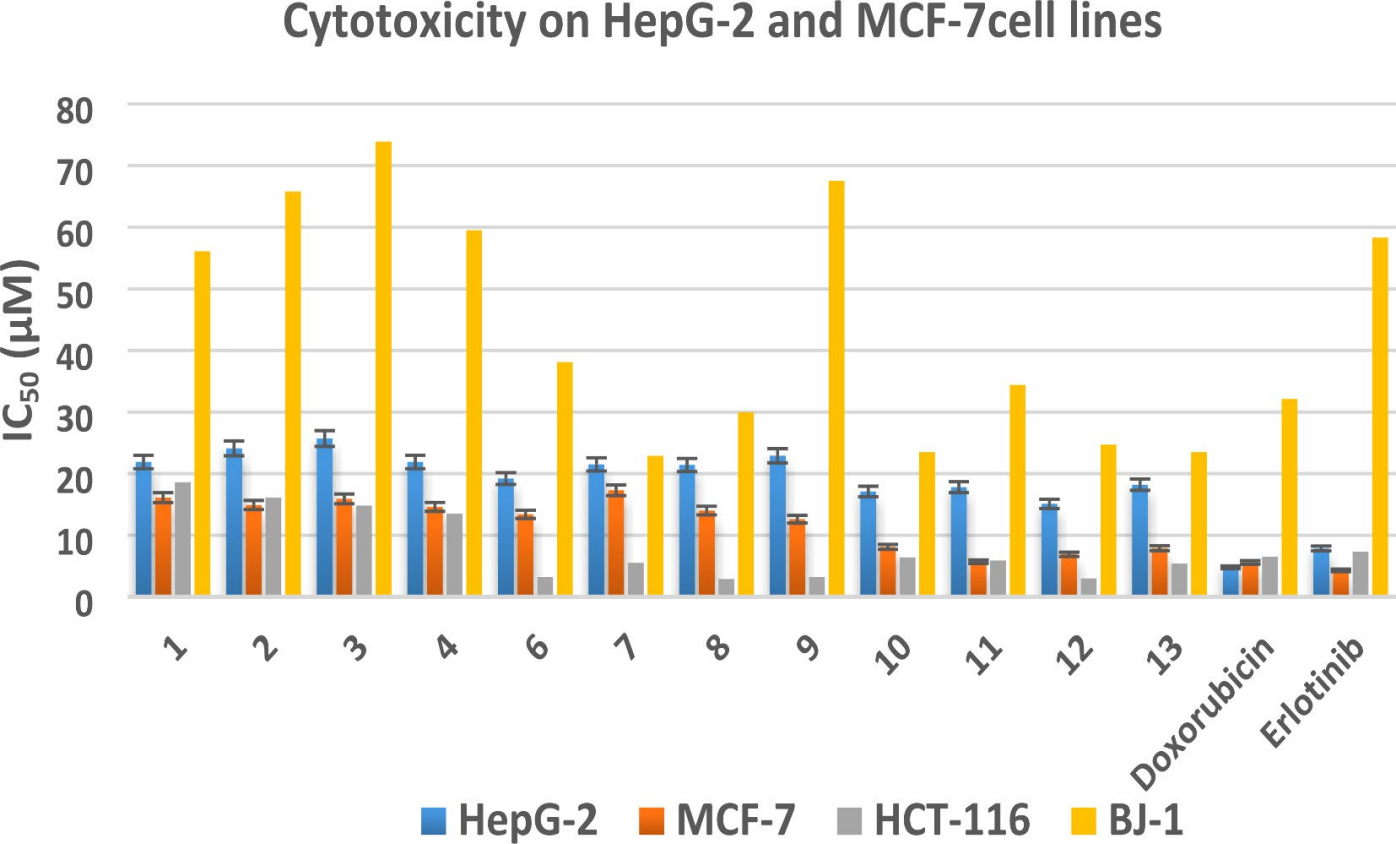
Cytotoxic action of quinazolinone-based targets **1**–**4** and **6**–**13** by means of MTT assay against human malignant HepG-2, MCF-7, HCT-116, and normal BJ-1cell lines.

The cytotoxic efficacy of quinazolin-4-one derivatives **1**–**4** and their derived 1,2,3-triazole glycosides **6**–**13** against human malignant liver HepG-2, breast MCF-7, colorectal HCT-116, and normal BJ-l cell lines generated from fibroblasts was shown in vitro via the MTT assay^48–50^ at several concentrations (25, 12.5, 6.25, and 3.125 µM) compared with doxorubicin and erlotinib (Figures S18–S21, supp. file), to determine their IC_50_, as presented in (Table S1, supp. file) and Fig. 2. All derivatives displayed weak cytotoxicity against HepG-2 cellular lines (IC_50 range_ = 15.10–25.70 µM) in contrast to doxorubicin and erlotinib (IC_50_ = 4.80 ± 0.50 and 7.8 ± 0.2 µM, respectively). Regarding to MCF-7, derivatives **1**–**4** and **6**–**9** revealed moderate activity (IC_50 range_ = 12.60–17.30 µM), while others **10**–**13** gave excellent ones (IC_50 range_ = 5.70–8.10 µM, IC_50 Doxorubicin_ = 5.6 ± 0.30 µM, IC_50 Erlotinib_ = 4.3 ± 0.1 µM). Additionally, quinazolinones **6**–**13** showed excellent and superior potency against HCT-116 (IC_50 range_ = 2.90–6.40 µM), however, others **1**–**4** exhibited moderate ones (IC_50 range_ = 13.50–18.60 µM, IC_50 Doxorubicin_ = 6.50 ± 0.50 µM, IC_50 Erlotinib_ = 7.3 ± 0.2 µM). The safety profiles of all derivatives were assessed through cytotoxic evaluation using human fibroblast-derived BJ-1 normal cell lines. Obtained results indicated that the derivatives were safe cytotoxic agents, having IC_50_ values that range from higher to moderate when compared to the reference (IC_50 range_ = 22.90–73.90 µM, IC_50 Doxorubicin_ = 32.10 ± 3.10 µM, IC_50 Erlotinib_ = 58.3 ± 1.4 µM).

#### Structure activity relationship study

**Fig. 3:**
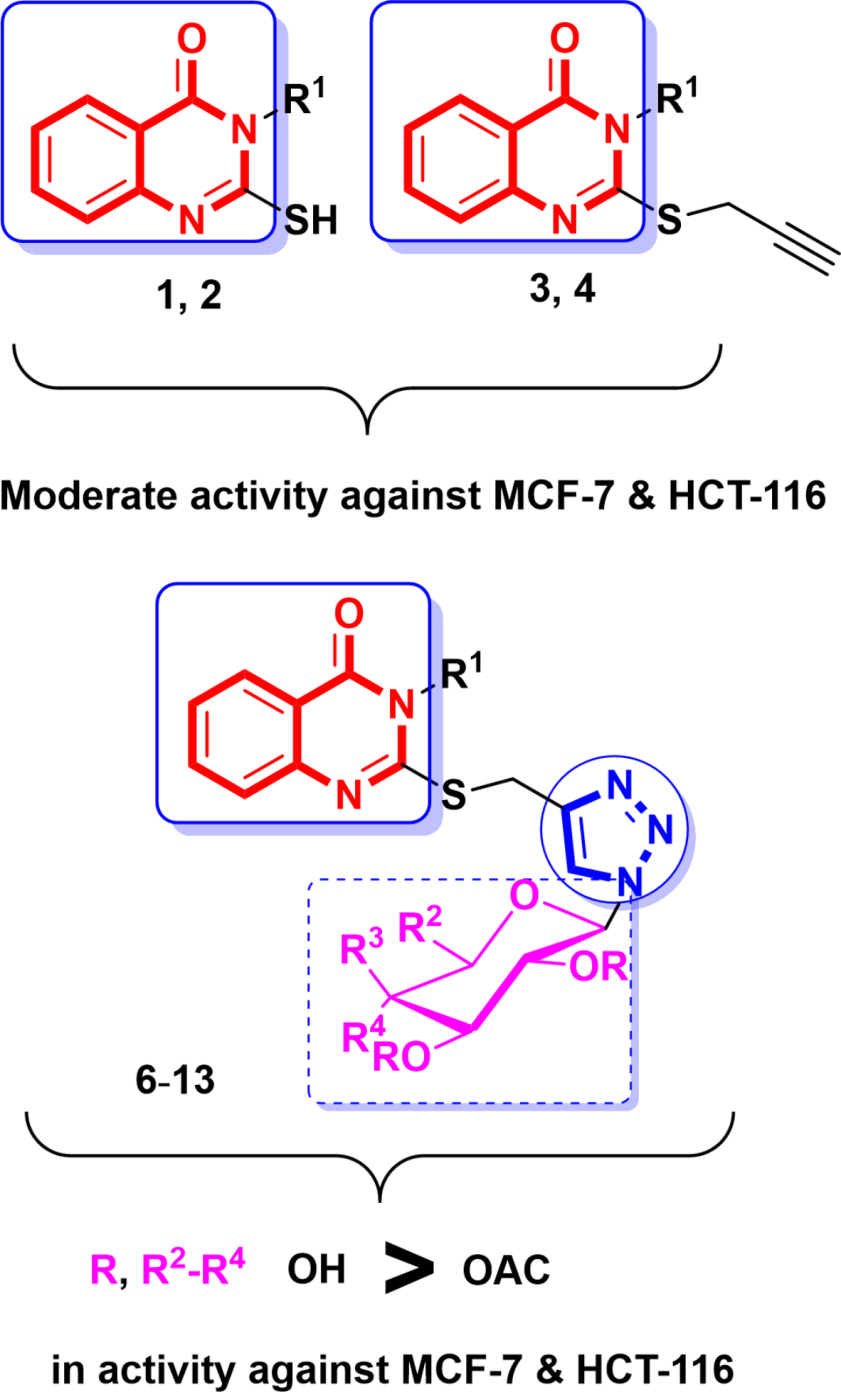
SAR study of quinazolin-4-ones **1**–**4** and **6**–**13** regarding to cytotoxicity against the MCF-7 and HCT-116 cell lines of human cancer.

Based on earlier findings displayed in (Table S1, supp. file) and Fig. 2, it was noted that 2-mercapto or 2-(prop-2-yn-1-ylthio)quinazolinones **1**–**4** displayed moderate cytotoxicity against HCT-116, MCF-7 cell lines (IC_50 range_ = 14.60–16.10 µM, 13.50–18.60 µM, respectively). Incorporation of quinazolinone core with acetylated glycosides and 1,2,3-triazole through thiomethyl linker in **6**–**9**, retain potency against MCF-7 with excellent and significant elevation against HCT-116 cell lines (IC_50 range_ = 12.60–17.30 µM, 2.90–5.50 µM, respectively). Alternatively, incorporation with hydroxylated glycosides in **10**–**13**, revealed excellent cytotoxicity against both cell lines (IC_50 range_ = 5.70–8.10 µM, 3.00–6.40 µM, in opposition to MCF-7 and HCT-116, respectively) (Fig. 3).

In conclusion, quinazolinone-based hybrids illustrated moderate potency against examined cancer cells MCF-7 and HCT-116, that enhanced against HCT-116 upon incorporation with 1,2,3-triazole-acetylated glycosides *via* a thiomethyl linker. Replacement with hydroxylated glycosides demonstrated encouraging and excellent efficacy contrary to both cell lines.

#### In vitro enzyme inhibitory evaluation against EGFR and VEGFR-2

The quinazolinone-based targets **6**–**13** were chosen for additional evaluation of their inhibitory potency in vitro against EGFR and VEGFR-2 in light of their remarkable cytotoxic outcomes, with the goal of elucidating their action mechanism. Table 1 depicts their IC_50_ values with erlotinib and sorafenib as standards, respectively^51,52^.

**Table 1:** Inhibitory in vitro results assessment of promising quinazolinone-1,2,3-triazole glycosides **6**–**13** relative to erlotinib and sorafenib, consequently, targeting EGFR and VEGFR-2.

Compound No.	IC_50_ (mean ± SD) (µM)	
	EGFR	VEGFR-2
**6**	18.63 ± 0.14	32.25 ± 0.25
**7**	17.94 ± 0.10	36.33 ± 0.17
**8**	21.38 ± 0.15	28.45 ± 0.50
**9**	23.05 ± 0.08	30.60 ± 0.32
**10**	16.75 ± 0.05	22.33 ± 0.10
**11**	0.35 ± 0.11	22.86 ± 0.13
**12**	15.88 ± 0.14	13.63 ± 0.08
**13**	0.31 ± 0.06	3.20 ± 0.15
**Erlotinib**	0.22 ± 0.05	–
**Sorafenib**	–	1.88 ± 0.10

*IC*_*50*_ Concentration of compound needed to 50% block the activity of the enzyme, *SD* Standard deviation; each value is the average of three values; (–) indicates no detection.

All screened quinazolinone revealed weak inhibitory activity against both enzymes (IC_50 range_ = 15.88–23.05 µM, 13.63–36.33 µM, against EGFR and VEGFR-2, respectively) contrasting with erlotinib and sorafenib (IC_50_ = 0.22 ± 0.05 and 1.88 ± 0.10 µM, respectively), except **11** and **13**. The latter hydroxylated glycosides with 3-methyl substitution of quinazolinone gave excellent potency against EGFR (IC_50_ = 0.35 ± 0.11 as well 0.31 ± 0.06 µM, in that order). Additionally, quinazolinone **13** devoted from hydroxymethyl fragment at p-6 of sugar moiety, exhibited extended excellent activity (IC_50_ = 3.20 ± 0.15 µM) against VEGFR-2.

#### Cell cycle arrest and apoptosis of compound 13

**Fig. 4:**
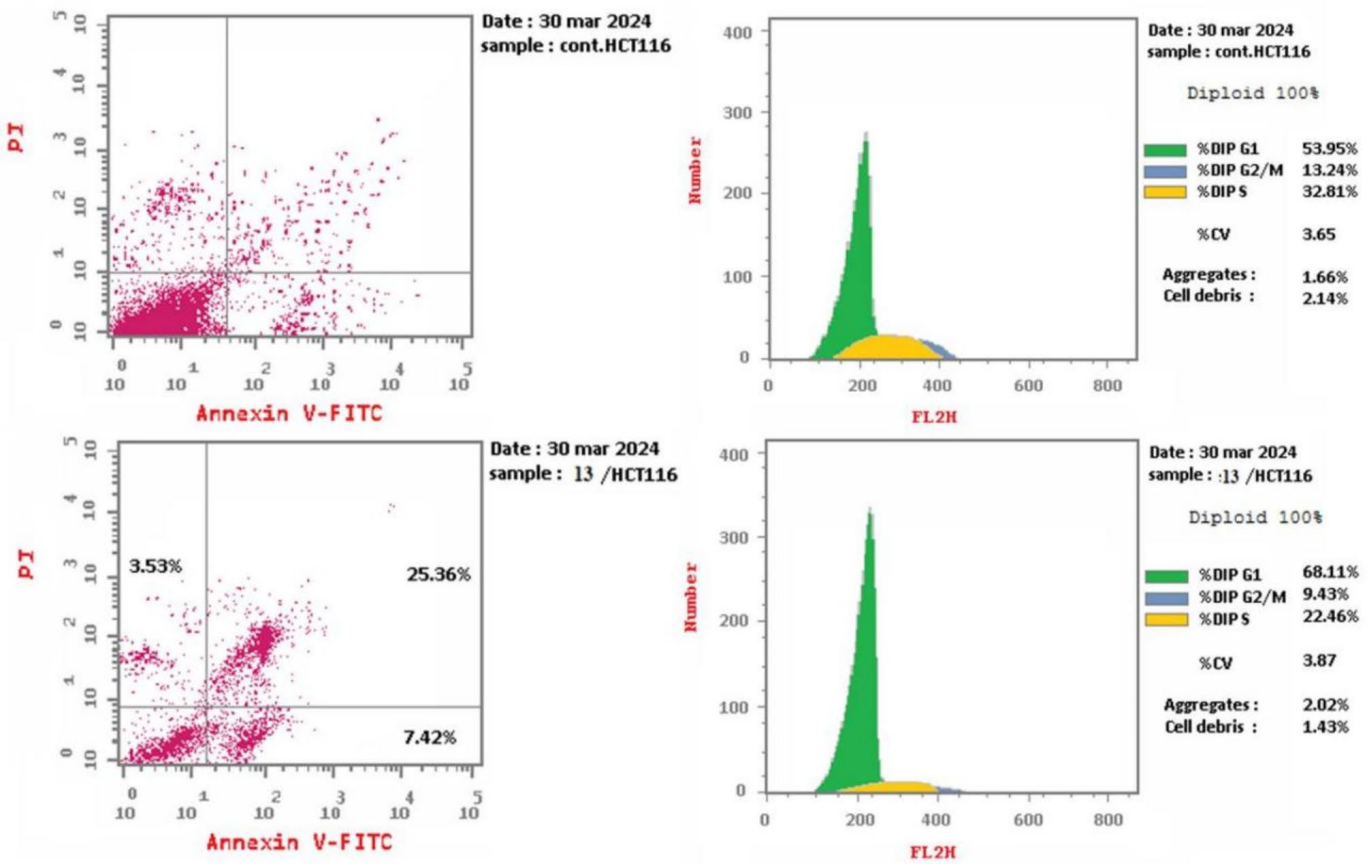
Analysis of the cellular cycle and impact of quinazolinone-1,2,3-triazole glycoside **13** on the proportion of V-FITC-positive annexin that stain in HCT-116 cells in comparison with control.

**Fig. 5:**
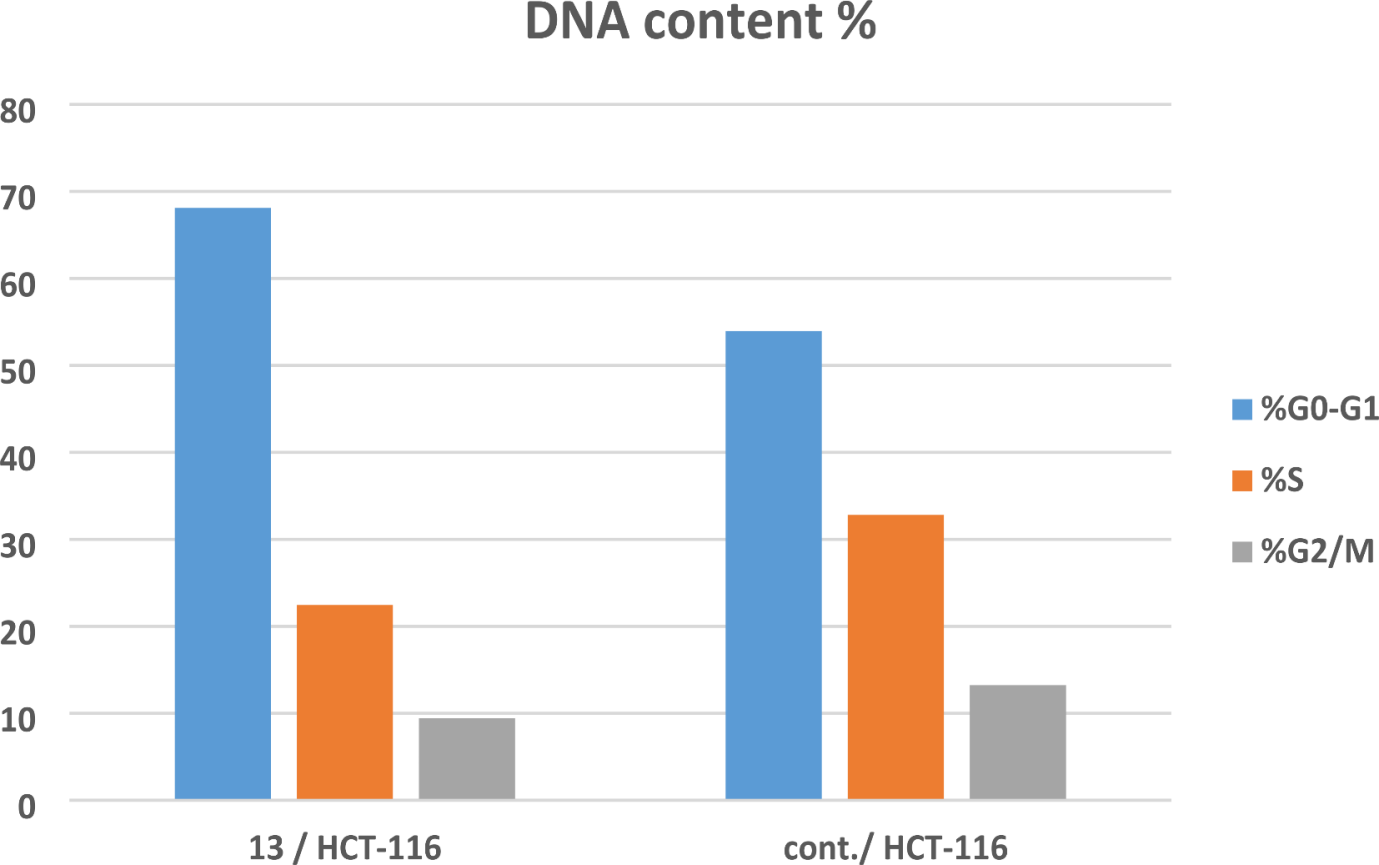
The cell cycle’s examination using quinazolinone-1,2,3-triazole glycoside **13**.

**Fig. 6:**
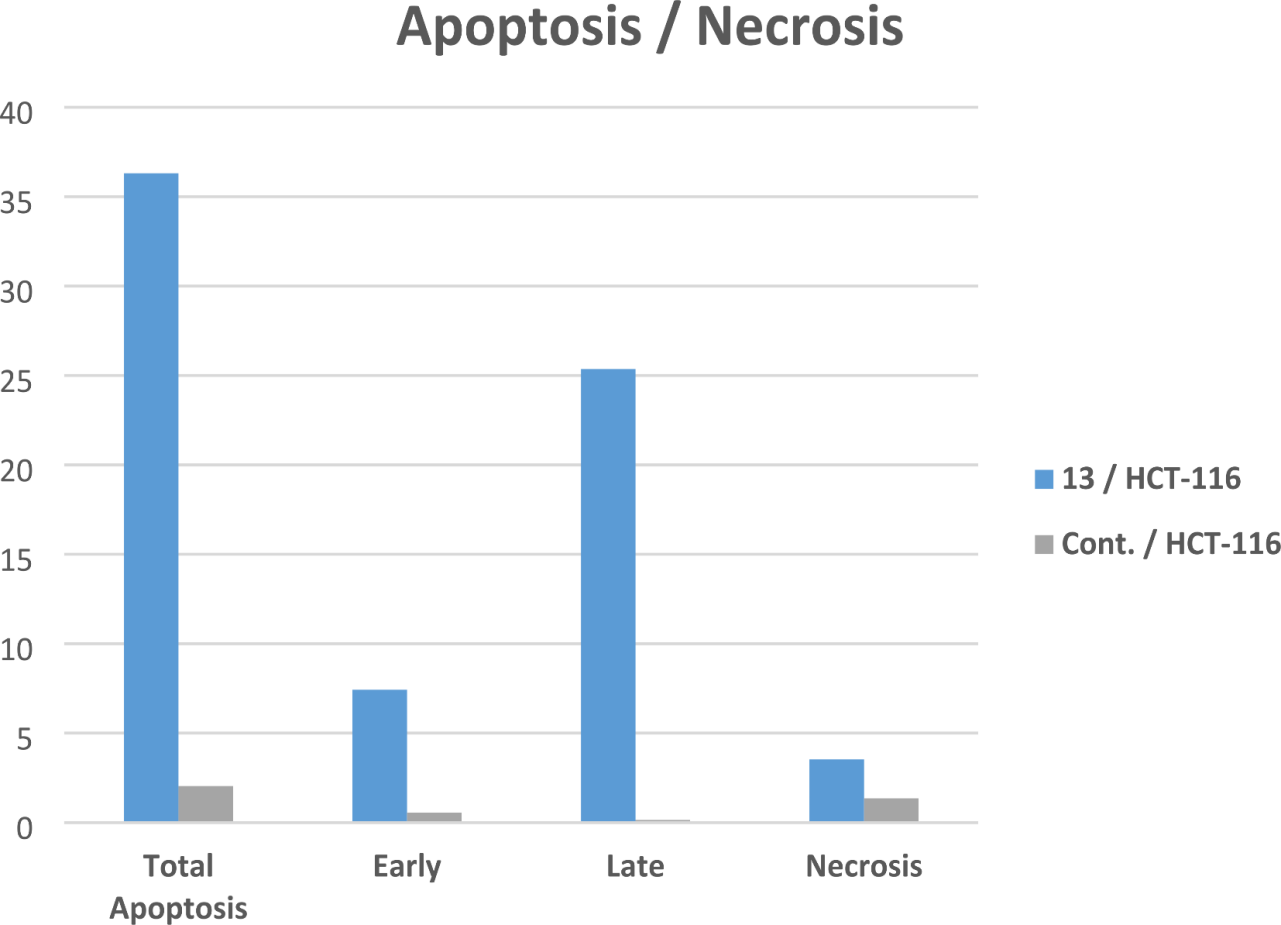
The effect of quinazolinone-1,2,3-triazole-glycoside **13** upon apoptosis.

The most powerful cytotoxic derivative, quinazolinone-1,2,3-triazole glycoside **13**, underwent 24-hour treatment at a dose of 5.40 µM to find out if it caused cell death by the apoptotic pathway in HCT-116 cells. Using annexin-V labeling, flow cytometry was employed to further analyze the cells (Figs. 4, 5 and 6).

As seen in Fig. 5 and (Table S2, supp. file), Target **13** showed greater accumulations of living cells of 68.11% during the G1 stage than did untreated MCF-7 tissue, which reported 53.95%. The data gathered made it abundantly evident that derivative **13** can stop HCT-116 cells in their G1 stage of the cell cycle.

With regard to apoptosis, the studied quinazolinone-1,2,3-triazole glycoside **13** caused a notable rise to 25.36% (for the later apoptosis from 0.14%) (DMSO control), and an extraordinary advancement to 7.42% in the preliminary phase of apoptosis from 0.55% (DMSO control) Fig. 6 and (Table S3 supp. file). Additionally, the derivative yielded a necrosis percentage of 3.53% compared to 1.35% for the DMSO control. As a result, compound **13** may trigger apoptosis, as indicated by a significant increase in apoptotic cells.

#### Effect of quinazolinone-1,2,3-triazole-glycoside 13 on Bax, Bcl-2, along with p53 levels within HCT-116 cells

The extrinsic pathway, regulated by the death receptor, and the intrinsic pathway, mediated by the mitochondria, are the two primary mechanisms that regulate the cell during apoptosis^53^. The two proteins can alter this planned process due to their respective roles as pro- and anti-apoptotic (inducer) Bcl-2 and Bax, and cell death is governed by balance between them^54^.

The tumor suppressor genetic code, p53, is another important factor that leads to cell death or inhibits cell proliferation. Cancers that retain their p53 suppression and genomic stability may proliferate more quickly and develop resistance to a variety of anticancer therapies^55^.

In contrast to control cells that have not been treated (33.85 Pg/mL), HCT-116 cells underwent a 24-hour treatment with compound **13**’s IC_50_ of 5.40 µM displayed in a 6.8-fold improvement in Bax genes values (229.40 Pg/ml). In contrast, compound **13** treatment reduced Bcl-2 protein levels in HCT-116 cells almost by 2.4 times, from 7.48 ng/mL to 3.15 ng/mL. Derivative **13** greatly enhanced p53 levels of proteins in treated HCT-116 cells by 6.3 times (885.72 Pg/mL) compared to in control cells (139.65 Pg/mL) (Table 2).

**Table 2:** Impact of quinazolinone-1,2,3-triazole-glycoside **13** affecting Bax, also known Bcl-2, along with p53 levels.

Compd.	Bax (Pg/mL)	Bcl-2 ng/mL	Bax/Bcl-2	P53 (Pg/mL)
**13 / HCT-116**	229.40 ± 1.62	3.15 ± 0.22	72.83	885.72 ± 1.49
**Cont./HCT-116**	33.85 ± 0.50	7.48 ± 0.10	4.53	139.65 ± 0.20

### In silico studies

#### ADMET prediction study

Analyzing absorption, distribution, metabolism, excretion, in addition to toxicity (ADMET) of targeted medications might give vital insights regarding an ideal therapeutic selection via the freely available internet-based applications SwissADME alongside admetSAR 1.0^56–58^. Both Veber’s and Lipinski’s rules are applicable to identify which medication is most effective when taken orally. As depicted in Table 3, quinazolinone-based targets **10**–**13** complied with the earlier rules with one violation to Veber rule (TPSA > 140), however derivatives **10** and **11** displayed additional violation to Lipinski’s principle (the number of N or O is greater than ten).

**Table 3:** Predicted physicochemical and lipophilic characteristics of the promising quinazolinone-1,2,3-triazole glycosides **10**–**13.**

Compd.	nRB^a^	nHBA^b^	nHBD^c^	TPSA (Å²)^d^	MW^e^	MLogP^f^	Violations^g^
**10**	6	9	4	181.05	497.52	0.72	1 (Lipinski); NorO > 10 1 (Veber); TPSA > 140
**11**	5	9	4	181.05	435.45	−0.85	1 (Lipinski); NorO > 10 1 (Veber); TPSA > 140
**12**	5	8	3	160.82	467.50	1.27	0 (Lipinski); 1 (Veber); TPSA > 140
**13**	4	8	3	160.82	405.43	−0.33	0 (Lipinski); 1 (Veber); TPSA > 140

^a^Rotatable bonds-number.

^b^Hydrogen bond Acceptor’s number.

^c^Hydrogen Bond Donor’s number.

^d^Topological Polar Surface Area.

^e^Molecular weight.

^f^Measurement of lipophilicity (MLog Po/w).

^g^Violations from Lipinski together with Veber rules.

**Fig. 7:**
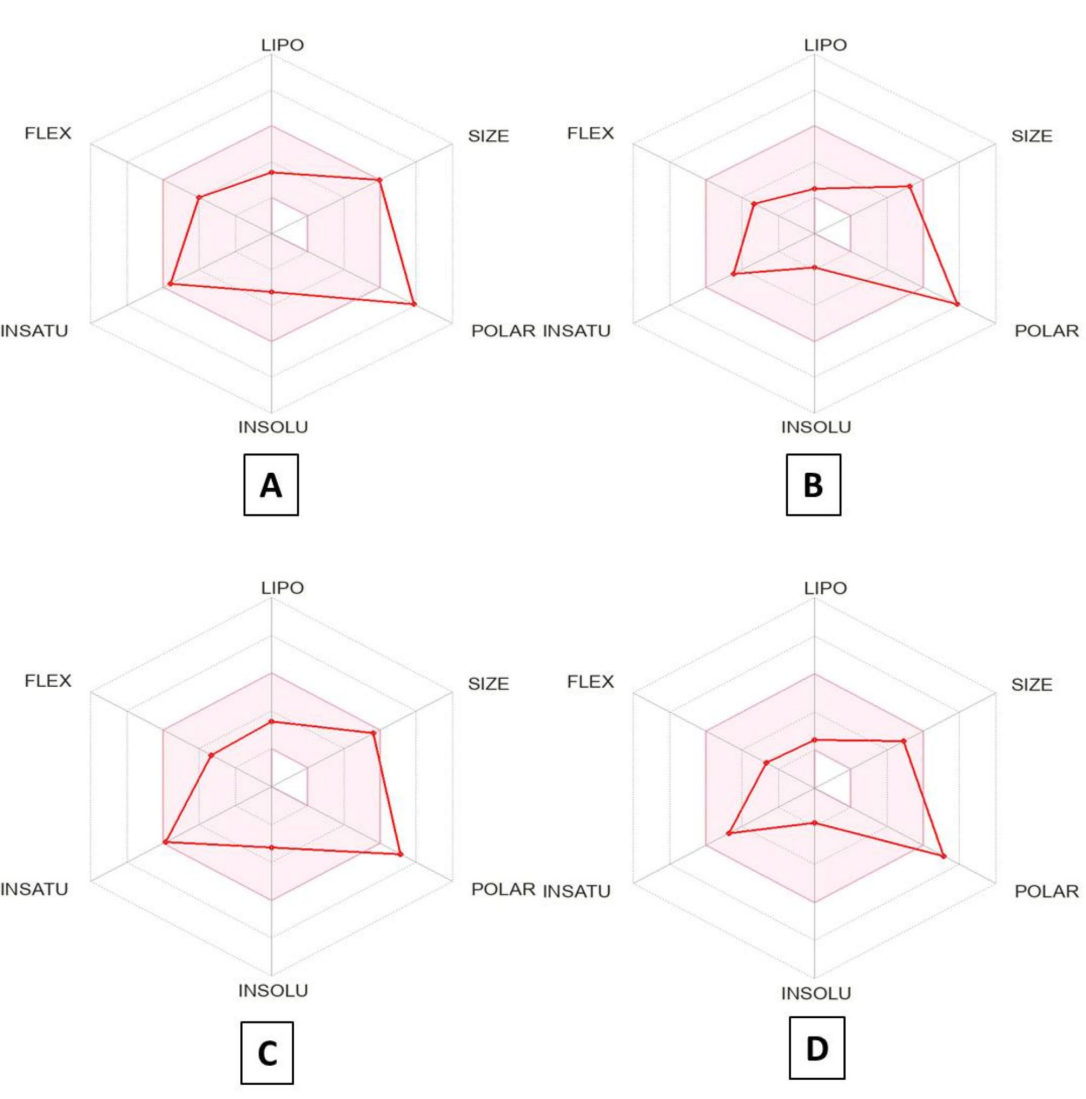
(**A**–**D**) indicates the powerful quinazolinone-1,2,3-triazole glycosides **10**–**13**’s radar chart of bioavailability, respectively. The anticipated results for the compounds under investigation have been indicated with red lines, and each element’s optimal values for bioavailability via the oral route were shown in the pink area.

The radar chart of bioavailability illustrates that the examined quinazolinones **10**–**13** were mentained outside of the optimal zone of polarity and inside the ideal range (pink region) for the remaining important factors (size, solubility, saturation, flexibility, and lipophilicity) (Fig. 7). Their oral biodegradability was well-indicated in these studies.

**Fig. 8:**
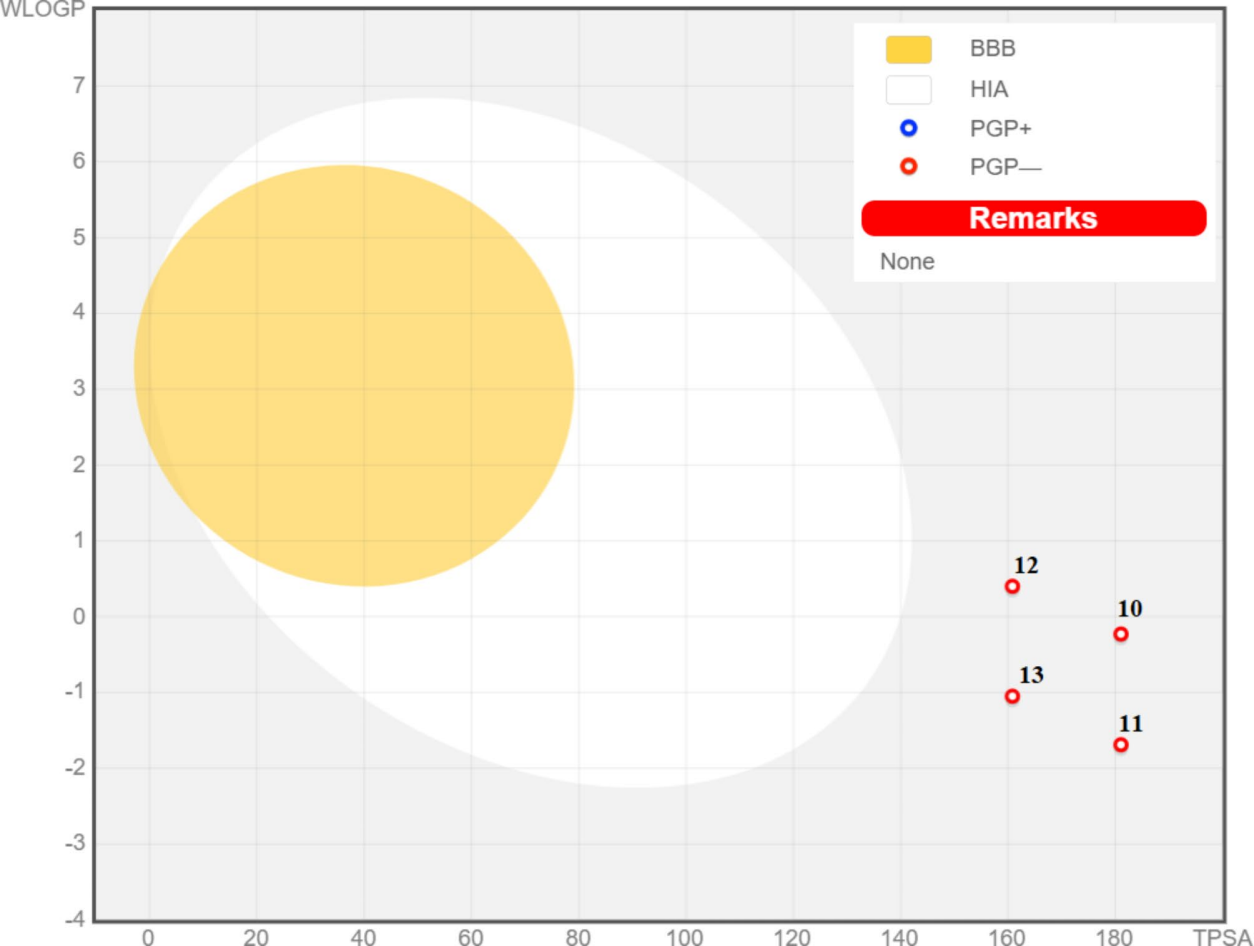
An image of a boiled egg illustrating how the powerful quinazolinone-1,2,3-triazole glycosides **10**–**13** can’t cross or enter gastrointestinal system and blood-brain barrier; PGP– is p-glycoprotein’s non-substrate form, and PGP + is its substrate form.

Figure 8 presents an analysis of the pharmacokinetic properties that may apply to quinazolinones **10**–**13**. All screened derivative was positioned away from the yellow and white zones on the Boiled-Egg chart. This implies that there was a low probability of gastrointestinal absorption with the absence of BBB penetration. As a result, these compounds may only be utilized for treating peripheral disorders.

Drug tolerance may be influenced by P-glycoprotein (P-gp), a drug efflux transporter that removes drugs from cells. The evaluated derivatives **10**–**13** are P-gp non-substrates (reddish dots displayed in Fig. 8), suggesting limited efflux out of the cell with the highest level of activity, according to the SwissADME website.

The potent quinazolinone-1,2,3-triazole glycosides **10**–**13** listed in (Table S4, supp. file), distributed and localized in mitochondria. Indeed, it is proven that if a certain molecule blocks more CYP enzymes, it may have a higher probability of participating drug-drug interactions (DDI) with other active compounds^59^. Therefore, it was expected that these compounds would be ineffective against these CYPs. Moreover, they did not show any of the predicted hazardous features, such as blockage of the potassium channel associated to the human ether-a-go-go gene (hERG). It means that cardiac adverse effects and cardiotoxicity—two main concerns in clinical trials with medication candidates—might not prove possible. On the other hand, all of the tested targets indicated signs of AMES toxicity, an important consideration early in the drug development process to determine whether those substances under study have the potential to be genotoxic.

These derivatives were categorized as harmless chemicals and placed in the third category based on inspections of severe oral toxicity. Furthermore, these substances may be classified considering it optional in addition to non-carcinogenic according to the carcinogenicity descriptor (CARC). It was anticipated that when evaluating biodegradation in the environment, all derivatives would be able to be recognized as non-biodegradable substances (Table S4, supp. file).

### Molecular docking simulation

According to the outstanding results from the evaluation of in vitro inhibitory assessment, docking of quinazolinone-1,2,3-triazole glycosides **11** and **13** with EGFR and VEGFR-2 was done. to find a relation between their activities and the possibility of binding mechanisms within the evaluated enzymes’ binding pockets. The molecular operating environment, or MOE-Dock, software, version 2014.0901 was implemented for docking procedures^60,61^. Firstly, the ligands that are native, sorafenib and erlotinib were re-docked into EGFR and VEGFR-2 binding sites (PDB IDs: 1M17 and 4ASD, respectively) to confirm the procedures^62–64^. As a result, the energy score values among the native ligands and their docked poses were − 11.51 and − 10.88 kcal/mol, along with fairly low RMSD readings (1.28 and 1.54 Å, in accordance).

**Fig. 9:**
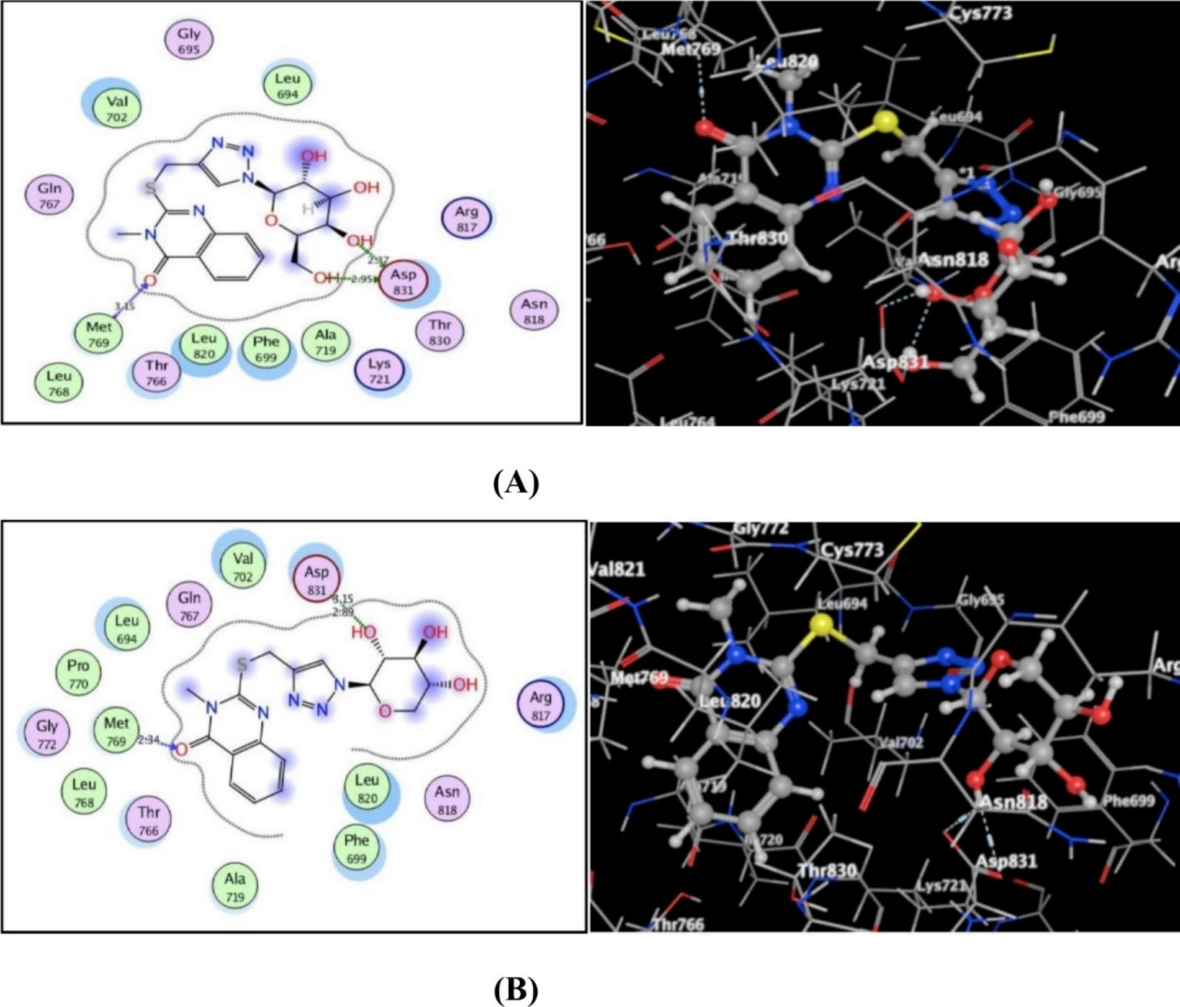
(**A**,**B**) sights displayed The quinazolinone-1,2,3-triazole glycosides **11** and **13**’s (2D and 3D) interactions within EGFR active site (PDB code: 1M17), correspondingly.

As depicted in Fig. 9, both quinazolinone analogues **11** and **13** were fitted well with the EGFR active site with promising energy being − 11.36 and − 11.45 kcal/mol, respectively. The carbonyl oxygen at p-4 of quinazolinone in **11** and **13** shared H-bond acceptor with the essential amino acid **Met769** backbone (distance: 3.15 and 2.34 Å, correspondingly). Also, sidechain of **Asp831** developed a hydrogen bond donors with sugar moieties’ hydroxyl oxygens (distance: 2.89, 3.15 Å in **13** and 2.37, 2.95 Å in **11**).

**Fig. 10:**
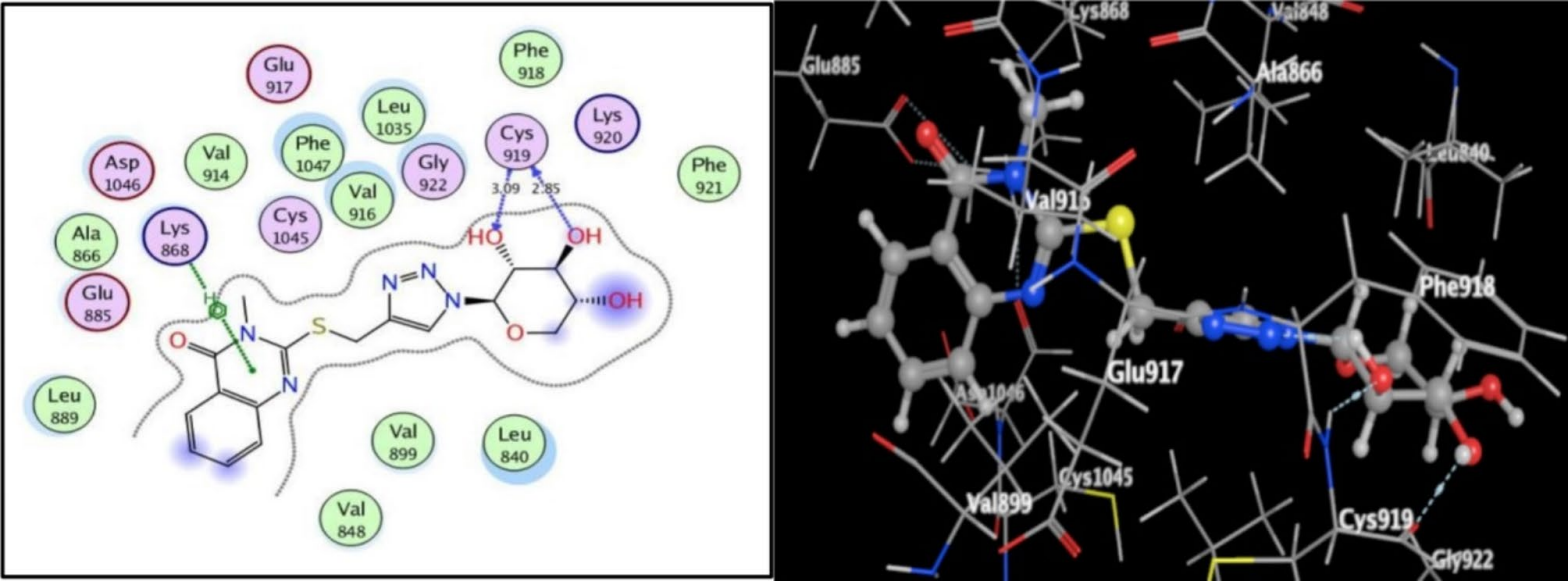
Quinazolinone-1,2,3-triazole glycoside **13**’s 2D and 3D pictures within the VEGFR-2 active site (PDB code: 4ASD, respectively).

On the other hand, quinazolinone-1,2,3-triazole glycoside **13** occupied the VEGFR-2 active site. The significant energy score found to be − 11.28 kcal/mol. Two hydroxyl oxygens of glycoside moiety afforded hydrogen bonding with **Cys919** backbone, which is the key amino acid (distance: 2.85 and 3.09 Å, correspondingly), similar to the sorafenib ligand. Additionally, arene-H interaction was observed between a quinazolinone core and **Lys868** (Fig. 10).

The quinazolinone scaffold with 3-methyl substitution that existed in compounds **11** and **13** and combined with hydroxylated glycosides via the 1,2,3-triazole moiety significantly fixed within EGFR active site via H-bonding formation with its crucial amino acid, **Met 769**. Hydroxymethyl fragment removal at p-6 of **13**’s glycoside core, facilitated its binding with the key amino acid **Cys919** within VEGFR-2 active site.

## Conclusion

The presented investigation displayed design and creation of a novel series of 1,2,3-triazolyl-glycosyl-qyunazoline hybrids with protected and free hydroxylated sugar moities *via* click chemistry strategy. A number of newly hybrid glycosides, in addition to their potent actitivy against cancer cells, revealed apparently a promising level of safety characteristics when compared to normal human cells. The deacetylated glycosyl-1,2,3-triazole structures 10–13 illustrated encouraging activities against MCF-7 (IC_50 range_ = 5.70–8.10 µM, IC_50 Doxorubicin_ = 5.6 ± 0.30 µM) whereas the free hydroxylated as well as their O-acetylated precoursors showed potent bahavoir against HCT-116 (IC_50 range_ = 2.90–6.40 µM). In addition, the promising candidate **13** was potent in inhibitory activity against EGFR and VEGFR-2 with significant IC_50_ comparable to the applied reference drugs. The quinazoline-based 1,2,3-triazole-glycoside **13**’s behavior toward apoptosis induction and cell cycle arrest activity findings also demonstrated its capacity to arrest MCF-7 cells within in their cell cycle’s G1 stage. Moreover, it was capable of upregulating the p53, Bax, and Bcl-2 stages throughout HCT-116 cells. Furthermore, molecular docking simulation was conducted to confirm a suggested mechanism of action and structural behavoir towards targeted enzymes. These findings are expected to prompt future research in medicinal chemistry concerning the chemotherapeutic field and lead to development of novel anticancer candidates with possible structural modifications.

## Experimental

### Chemistry

Synthesis of compounds **1**, **2** and **3** were prepared as earlier reported procedure ^65,66^ respectively, ^1^H,^13^C-NMR spectra for new compounds attatched in supp. file Figs. S1–S17.

#### Synthesis of 3-methyl-2-(prop-2-yn-1-ylthio)quinazolin-4(3 H)-one.(4)

After stirring a solution containing 2 mmol of thiol derivatives **2** and potassium hydroxide into 20 mL of DMF for half an hour at the ambient temperature, the mixture was chilled to 0 °C. Propargyl bromide (2.2 mmol) put in mixture gradually at zero degree for 15 min, then came agitated for a duration of six hours at ambient temperature. TLC tracked the reaction’s progression utilizing a solvent blend of ethyl acetate and petroleum ether in a 1:3 proportion. Icy-cold water was added while shaking constantly, followed by filtration of the resultant solid precipitate, washing with cold ethanol, and drying to get acetylenic compound **4**.

Yield: 93%; m.p. 163–165 °C; IR (KBr) cm^− 1^, *ν*: 3255 (alkyne-CH), 3099 (aromatic C-H), 1756 (C = O); ^1^H-NMR (500 MHz, CDCl_3_) *δ*/ppm: 8.22 (dd, 1H, *J* = 8.0, 1.6 Hz, Ar-H), 7.71 (dd, 1H, *J* = 8.5, 7.0 Hz, Ar-H), 7.64 (d, 1H, *J* = 8.2 Hz, Ar-H), 7.41 (dd, 1H, *J* = 8.1, 7.1 Hz, Ar-H), 4.16 (d, 2 H, *J* = 2.7 Hz, CH2), 3.61 (s, 3 H, CH3), 2.27 (t, 1H, *J* = 2.6 Hz, CH). ^13^C NMR (125 MHz, CDCl_3_) *δ* 161.85 (C = O), 155.19, 147.34, 134.40, 127.09, 126.29, 126.02, 119.27 (Ar-C), 78.45 (≡ C), 71.78 (≡ CH), 30.27 (CH_3_), 20.90 (S-CH_2_). Analysis calcd. for C_12_H_10_N_2_OS (230.29): C, 62.59; H, 4.38; N, 12.16. Found: C, 62.63; H, 4.26; N, 12.20.%.

#### Synthesis of targeted 1,2,3-triazole substituted-quinazolin hybrid acetylated glycosides 6–9

2,3,4-tri-*O*-acetyl-*d*-xylopyranosyl or 2,3,4,6-tetra-*O*-acetyl-*d*-galactopyranosyl azide (2.0 mmol) was combined with a deep well -agitated solution of the derivative of terminal alkyne **3** or **4** (2.0 mmol) in a 2/1 THF/H_2_O mixture of solvents (15 mL). Initially, Diisopropylethylamine (DIPEA) in a catalytic amount (three drops) and sodium ascorbate (0.4 mmol, 0.08 g) were added, then (0.4 mmol, 0.11 g) of copper sulfate pentahydrate was incorporated. During a 24-hour period, the r.m. was swirled at ambient temperature under TLC observation using a pet ether/ethyl acetate (4:1) solvent solution. Two aliquots of ethyl acetate (30 mL each) were introduced into the mixture, and the isolation of the organic phase was occurred. The layers of organic material were gathered, dehydrated using anhydrous sodium sulfate, and subsequently vaporized. Triazole glycosides **6**–**9** were isolated by additional purification utilizing column chromatography with a solvent mixture of hexane/ethyl acetate (5:1).

#### 2-(((1-[2,3,4,6-Tetra-O-acetyl-β-D-galactopyranosyl]-1 H-1,2,3-triazol-4-yl)methyl)thio)-3-phenylquinazolin-4(3 H)-one.(6)

Yield: 87%; m.p. 90–92 °C; IR (KBr) cm^− 1^, *ν*: 3100 (aromatic C-H), 1752 (C = O); ^1^H-NMR (500 MHz, CDCl_3_) *δ*/ppm: 8.27 (s, 1H, Ar-H), 8.09 (d, 1H, *J* = 7.9 Hz, Ar-H), 7.86 (t, 1H, *J* = 7.7 Hz, Ar-H), 7.72 (d, 1H, *J* = 8.6 Hz, Ar-H), 7.58–7.55 (m, 3 H, Ar-H), 7.51–7.48 (m, 2 H, Ar-H), 7.44 (d, *J* = 4.8 Hz, 1H, Ar-H), 6.20 (d, 1H, *J* = 9.2 Hz, H-1′ _anomeric_), 5.53 (t, 1H, *J* = 9.5 Hz, H-3′), 5.43 (d, 1H, *J* = 3.5 Hz, H-2′), 5.40 (dd, *J* = 4.1, 2.9 Hz, 1H, H-4′), 4.56–4.54 (m, 1H, H-5′), 4.46 (s, 2 H, CH2), 4.10 (dd, *J* = 11.6, 4.9 Hz, 1H, H-6′), 3.99 (d, *J* = 9.5 Hz, 1H, H-6′′), 2.15, 1.96, 1.93, 1.71 (4s, 12 H, CH_3_CO). ^13^C NMR (125 MHz, CDCl_3_) *δ* 169.98, 169.52, 168.49 (4 C = O acetyl), 160.78 (C = O), 156.43 (N = C-N), 147.21, 143.21, 135.73, 134.98, 130.01, 129.57, 129.41, 126.64, 126.14, 123.36, 119.64 (Ar-C), 84.17 (C-1_anomeric_),, 74.03 (C-5), 72.94 (C-3), 70.35 (C-4), 67.67 (C-2), 61.61 (C-6), 26.53 (S-CH_2_), 20.50, 20.42, 20.35, 19.88 (4CH_3_ acetyl). Analysis calcd. for C_31_H_31_N_5_O_10_S (665.67): C, 55.93; H, 4.69; N, 10.52. Found: C, 56.01; H, 4.59; N, 10.61.%.

#### 2-(((1-[2,3,4,6-Tetra-O-acetyl-β-D-galactopyranosyl]-1 H-1,2,3-triazol-4-yl)methyl)thio)-3-methylquinazolin-4(3 H)-one.(7)

Yield: 84%; m.p. 140–142 °C; IR (KBr) cm^− 1^, *ν*: 3110 (aromatic C-H), 1753 (C = O); ^1^H-NMR (500 MHz, CDCl_3_) *δ*/ppm: 8.32 (s, 1H, Ar-H), 8.08 (d, 1H, *J* = 9.7 Hz, Ar-H), 7.81 (t, 1H, *J* = 6.9 Hz, Ar-H), 7.66 (d, 1H, *J* = 8.1 Hz, Ar-H), 7.47–7.44 (m, 1H, Ar-H), 6.22 (d, 1H, *J* = 9.1 Hz, H-1′ _anomeric_), 5.54 (t, 1H, *J* = 9.5 Hz, H-3′), 5.43 (d, 1H, *J* = 3.3 Hz, H-2′), 5.41–5.39 (m, 1H, H-4′), 4.61 (s, 2 H, CH_2_), 4.54 (t, 1H, *J* = 6.3 Hz, H-5′), 4.09 (dd, *J* = 11.5, 4.9 Hz, 1H, H-6′), 3.97 (dd, *J* = 11.6, 7.3 Hz, 1H, H-6′′), 3.49 (s, 3 H, CH_3_), 2.15, 1.95, 1.93, 1.71 (4s, 12 H, CH_3_CO). ^13^C NMR (125 MHz, CDCl_3_) *δ* 169.93, 169.49, 168.45 (4 C = O acetyl), 160.72 (C = O), 156.24 (N = C-N), 146.76, 143.20, 134.55, 126.43, 125.90, 123.46, 118.67 (Ar-C), 84.18 (C-1_anomeric_), 72.92 (C-5), 70.36 (C-3), 67.67 (C-4), 67.33 (C-2), 61.60 (C-6), 30.07 (CH_3_), 26.12 (S-CH_2_), 20.47, 20.40, 20.34, 19.85 (4CH_3_ acetyl). Analysis calcd. for C_26_H_29_N_5_O_10_S (603.60): C, 51.74; H, 4.84; N, 11.60. Found: C, 51.81; H, 4.79; N, 11.65.%.

#### 2-(((1-[2,3,4-tri-O-acetyl-β-D-xylopyranosyl]-1 H-1,2,3-triazol-4-yl)methyl)thio)-3-phenylquinazolin-4(3 H)-one.(8)

Yield: 86%; m.p. 210–212 °C; IR (KBr) cm^− 1^, *ν*: 3079 (aromatic C-H), 1746 (C = O); ^1^H-NMR (500 MHz, CDCl_3_) *δ*/ppm: 8.33 (s, 1H, Ar-H), 8.09 (d, 1H, *J* = 7.9 Hz, Ar-H), 7.86 (t, 1H, *J* = 7.7 Hz, Ar-H), 7.70 (d, 1H, *J* = 8.2 Hz, Ar-H), 7.56 (dd, 3 H, *J* = 5.2, 1.9 Hz, Ar-H), 7.49 (t, 1H, *J* = 6.9 Hz, Ar-H), 7.46–7.44 (m, 2 H, Ar-H), 6.16 (d, 1H, *J* = 9.1 Hz, H-1′ _anomeric_), 5.56 (t, 1H, *J* = 9.3 Hz, H-2′), 5.45 (t, *J* = 9.5 Hz, 1H, H-3′), 5.10 (td, *J* = 10.0, 5.6 Hz, 1H, H-4′), 4.46 (s, 2 H, CH2), 4.05 (dd, *J* = 11.3, 5.6 Hz, 1H, H-5′), 3.80 (d, *J* = 10.9 Hz, 1H, H-5′′), 2.01, 1.97, 1.66 (3s, 9 H, CH_3_CO). ^13^C NMR (125 MHz, CDCl_3_) *δ* 169.61, 168.42 (3 C = O acetyl), 160.79 (C = O), 156.31 (N = C-N), 147.24, 143.52, 135.74, 134.93, 129.99, 129.55, 129.45, 126.56, 126.32, 126.10, 122.66, 119.63 (Ar-C), 84.62 (C-1_anomeric_), 71.88 (C-3), 70.14 (C-2), 67.97 (C-4), 64.11 (C-5), 26.60 (S-CH_2_), 20.49, 20.36, 19.78 (3CH_3_ acetyl). Analysis calcd. for C_28_H_27_N_5_O_8_S (593.61): C, 56.65; H, 4.58; N, 11.80. Found: C, 56.56; H, 4.47; N, 11.86.%.

#### 2-(((1-[2,3,4-tri-O-acetyl-β-D-xylopyranosyl]-1 H-1,2,3-triazol-4-yl)methyl)thio)-3-methylquinazolin-4(3 H)-one.(9)

Yield: 85%; m.p. 146–148 °C; IR (KBr) cm^− 1^, *ν*: 3094 (aromatic C-H), 1757 (C = O); ^1^H-NMR (500 MHz, CDCl_3_) *δ*/ppm: 8.38 (s, 1H, Ar-H), 8.08 (d, 1H, *J* = 8.0 Hz, Ar-H), 7.82–7.78 (m, 1H, Ar-H), 7.63 (d, 1H, *J* = 8.4 Hz, Ar-H), 7.45 (td, 1H, *J* = 7.9, 3.4 Hz, Ar-H), 6.17 (d, 1H, *J* = 9.2 Hz, H-1′ _anomeric_), 5.57 (t, 1H, *J* = 9.3 Hz, H-2′), 5.45 (t, *J* = 9.5 Hz, 1H, H-3′), 5.09 (dt, *J* = 10.3, 5.2 Hz, 1H, H-4′), 4.59 (s, 2 H, CH2), 4.09–4.03 (m, 1H, H-5′), 3.79 (d, *J* = 11.3 Hz, 1H, H-5′′), 3.49 (s, 3 H, CH3), 2.00, 1.97, 1.67 (3s, 9 H, CH_3_CO). ^13^C NMR (125 MHz, CDCl_3_) *δ* 169.63, 168.44 (3 C = O acetyl), 160.77 (C = O), 156.13 (N = C-N), 146.80, 143.50, 134.55, 126.39, 126.12, 125.90, 122.86, 120.71, 118.67 (Ar-C), 84.66 (C-1_anomeric_), 71.91 (C-3), 70.17 (C-2), 67.99 (C-4), 64.14 (C-5), 30.08 (CH_3_), 26.22 (S-CH_2_), 20.51, 20.38, 19.81 (3CH_3_ acetyl). Analysis calcd. for C_23_H_25_N_5_O_8_S (531.54): C, 51.97; H, 4.74; N, 13.18. Found: C, 55.05; H, 4.79; N, 13.09.%.

#### Synthesis of targeted 1,2,3-triazole substituted-quinazolin hybrid de-acetylated glycosides10-13

A solution of compounds **6–9** (2 mmol) underwent agitation for 24 h at ambient temperature in a combination of dry methanol (25 mL) and saturated gaseous ammonia. The solvent was evaporated within a temperature range of 40–45 °C. The ppt. dissolution occurred in ethanol at a temperature of 40 °C and kept for two hours at ambient temperature. The solvent was reduced under reduced pressure, and the resultant solid was filtered and dried then subjected to column chromatography (pet. ether : ethyl acetate; 3:1) to give compounds **10–13**.

#### 2-(((1-[β-D-Galactopyranosyl]-1 H-1,2,3-triazol-4-yl)methyl)thio)-3-phenylquinazolin-4(3 H)-one.(10)

Yield: 61%; m.p. 163–165 °C; IR (KBr) cm^− 1^, *ν*: 3440–3420 (OH), 3065 (C-H), 1752 (C = O); ^1^H-NMR (500 MHz, DMSO-d_6_) *δ*/ppm: 8.20 (s, 1H, Ar-H), 8.10 (t, 1H, *J* = 6.1 Hz, Ar-H), 7.88–7.84 (m, 1H, Ar-H), 7.73 (dd, 1H, *J* = 8.2, 4.2 Hz, Ar-H), 7.55 (d, 3 H, *J* = 4.9 Hz, Ar-H), 7.48 (d, 3 H, *J* = 5.1 Hz, Ar-H), 5.43 (d, 1H, *J* = 9.3 Hz, H-1′ _anomeric_), 5.21–5.19 (m, 1H, OH), 5.03-5.00 (m, 1H, OH), 4.69–4.65 (m, 1H, OH), 4.63–4.61 (m, 1H, OH), 4.49 (s, 2 H, CH2), 4.00-3.96 (m, 2 H, H-2′, H-3′), 3.74 (t, *J* = 4.7 Hz, 1H, H-4′), 3.67 (d, 1H, *J* = 5.6, H-5′), 3.52–3.48 (m, 2 H, H-6′′, H-6′). ^13^C NMR (125 MHz, DMSO-*D*_6_) *δ* 160.80 (C = O), 156.64 (N = C-N), 147.30, 142.11, 135.79, 135.00, 130.01, 129.57, 129.45, 126.60, 126.29, 126.09, 123.14, 119.65 (Ar-C), 88.11 (C-1_anomeric_), 77.05 (C-5), 71.88 (C-3), 69.11 (C-4), 68.30 (C-2), 60.45 (C-6), 26.75 (S-CH_2_). Analysis calcd. for C_23_H_23_N_5_O_6_S (497.53): C, 55.53; H, 4.66; N, 14.08. Found: C, 55.57; H, 4.70; N, 13.99.%.

#### 2-(((1-[β-D-Galactopyranosyl]-1 H-1,2,3-triazol-4-yl)methyl)thio)-3-methylquinazolin-4(3 H)-one.(11)

Yield: 58%; m.p. 141–143 °C; IR (KBr) cm^− 1^, *ν*: 3439–3418 (OH), 3062 (C-H), 1750 (C = O); ^1^H-NMR (500 MHz, DMSO-d_6_) *δ*/ppm: 8.24 (s, 1H, Ar-H), 8.08 (d, 1H, *J* = 7.9 Hz, Ar-H), 7.80 (t, *J* = 7.6 Hz, 1H, Ar-H), 7.65 (d, 1H, *J* = 8.2 Hz, Ar-H), 7.46 (t, 1H, *J* = 7.8 Hz, Ar-H), 5.46 (d, 1H, *J* = 9.4 Hz, H-1′ _anomeric_), 5.23–5.22 (m, 1H, OH), 5.03–5.01 (m, 1H, OH), 4.69–4.66 (m, 1H, OH), 4.64 (s, 2 H, CH2), 4.03–3.96 (m, 2 H, H-3′, OH), 3.74 (t, 1H, *J* = 4.2 Hz, H-2′), 3.69 (t, 1H, *J* = 6.3 Hz, H-4′), 3.54–3.51 (m, 1H, H-5′), 3.49 (s, 3 H, CH3), 3.46–3.45 (m, 1H, H-6′), 3.39–3.38 (m, 1H, H-6′′). ^13^C NMR (125 MHz, DMSO-*D*_6_) *δ* 160.72 (C = O), 156.38 (N = C-N), 146.74, 142.25, 134.58, 126.51, 126.40, 125.98, 125.88, 124.36, 122.97, 118.58 (Ar-C), 88.09 (C-1_anomeric_), 78.42 (C-5), 73.70 (C-3), 69.21 (C-4), 68.44 (C-2), 60.45 (C-6), 30.02 (CH_3_), 26.29 (S-CH_2_). Analysis calcd. for C_18_H_21_N_5_O_6_S (435.46): C, 49.65; H, 4.86; N, 16.08. Found: C, 49.72; H, 4.90; N, 15.98.%.

#### 2-(((1-[β-D-Xylopyranosyl]-1 H-1,2,3-triazol-4-yl)methyl)thio)-3-phenylquinazolin-4(3 H)-one.(12)

Yield: 60%; m.p. 158–160 °C; IR (KBr) cm^− 1^, *ν*: 3440–3420 (OH), 3065 (C-H), 1752 (C = O); ^1^H-NMR (500 MHz, DMSO-d_6_) *δ*/ppm: 8.24 (s, 1H, Ar-H), 8.10 (d, 1H, *J* = 7.9 Hz, Ar-H), 7.86 (t, 1H, *J* = 7.7 Hz, Ar-H), 7.73 (d, 1H, *J* = 8.2 Hz, Ar-H), 7.55 (d, 3 H, *J* = 5.5 Hz, Ar-H), 7.50 (t, 1H, *J* = 7.5 Hz, Ar-H), 7.47–6.45 (m, 2 H, Ar-H), 5.42 (d, 1H, *J* = 9.3 Hz, H-1′ _anomeric_), 5.37–5.36 (m, 1H, OH), 5.30–5.29 (m, 1H, OH), 5.16–5.15 (m, 1H, 1OH), 4.47 (s, 2 H, CH2), 3.79 (dd, 1H, *J* = 11.2, 5.3 Hz H-2′), 3.72 (td, 1H, *J* = 9.0, 6.0 Hz, H-3′), 3.43 (dq, 1H, *J* = 10.2, 5.1 Hz, H-4′), 3.32–3.27 (m, 2 H, H-5′′, H-5*’*). ^13^C NMR (125 MHz, DMSO-*D*_6_) *δ* 160.79 (C = O), 156.63 (N = C-N), 147.26, 142.13, 135.75, 134.98, 130.00, 129.56, 129.46, 126.61, 126.28, 126.11, 123.13, 119.65 (Ar-C), 88.10 (C-1_anomeric_), 77.06 (C-4), 71.89 (C-3), 69.11 (C-2), 68.31 (C-5), 26.76 (S-CH_2_). Analysis calcd. for C_22_H_21_N_5_O_5_S (467.50): C, 56.52; H, 4.53; N, 14.98. Found: C, 56.60; H, 4.47; N, 15.03.%.

#### 2-(((1-[β-D-Xylopyranosyl]-1 H-1,2,3-triazol-4-yl)methyl)thio)-3-methylquinazolin-4(3 H)-one.(13)

Yield: 59%; m.p. 216–218 °C; IR (KBr) cm^− 1^, *ν*: 3442–3427 (OH), 3071 (C-H), 1753 (C = O); ^1^H-NMR (500 MHz, DMSO-d_6_) *δ*/ppm: 8.28 (s, 1H, Ar-H), 8.09 (dd, 1H, *J* = 7.9, 1.6 Hz, Ar-H), 7.82–7.79 (m, 1H, Ar-H), 7.66 (d, 1H, *J* = 8.1 Hz, Ar-H), 7.46 (td, 1H, *J* = 7.6 Hz, Ar-H), 5.45 (d, 1H, *J* = 9.3 Hz, H-1′ _anomeric_), 5.38–5.37 (m, 1H, OH), 5.29–5.28 (m, 1H, OH), 5.15–5.14 (m, 1H, OH), 4.62 (s, 2 H, CH2), 3.79 (dd, 1H, *J* = 11.1, 5.3 Hz H-2′), 3.73 (d, 1H, H-3′), 3.45–3.42 (m, 1H, H-4′), 3.33 (s, 3 H, CH3), 3.32–3.28 (m, 2 H, H-5′′, H-5*’*). ^13^C NMR (125 MHz, DMSO-*D*_6_) *δ* 160.72 (C = O), 156.36 (N = C-N), 146.78, 142.29, 134.56, 126.40, 126.05, 125.87, 123.20, 118.64 (Ar-C), 88.08 (C-1_anomeric_), 77.08 (C-4), 71.90 (C-3), 69.10 (C-2), 68.31 (C-5), 30.03 (CH_3_), 26.27 (S-CH_2_). Analysis calcd. for C_17_H_19_N_5_O_5_S (405.43): C, 50.36; H, 4.72; N, 17.27. Found: C, 50.41; H, 4.79; N, 17.19.%.

### Cytotoxic activity

The recently incorporated quinazolinone-based targets **1**–**4**,** 6**–**13** underwent evaluation for their cytotoxic effects with in vitro employing MTT technique, according to the procedure that has been published^48–50^, utilizing human fibroblast-derived BJ-1 normal cell line, and human cancer liver HepG-2, breast MCF-7, and colon HCT-116 cell lines. These cell lines were purchased from Karolinska Center, Department of Oncology and Pathology, Karolinska Institute and Hospital, Stockholm, Sweden, There were further details in the supplemental file.

#### Inhibitory evaluation against EGFR and VEGFR-2 activities

The optimistic quinazolinone-based derivatives **6**–**13** were evaluated for their capability to inhibit EGFR as well as VEGFR-2 activities in vitro, following the technique described earlier and employing erlotinib and sorafenib as references^51,52^. There was further data in the supplemental file.

#### **Exploration of apoptosis and cell cycle investigation with compound 13**

The examination of apoptosis and the interpretation of the cell cycle were described^37^ utilizing flow cytometry. HCT-116 cells were treated with quinacolinone-1,2,3-triazole glycoside **13** for a full day, and after that, the cells were cultured at 37 °C. There were more details added to supporting documentation .

#### The influence of compound 13 on HCT-116 cell levels of p53, Bax and Bcl-2

The methods previously described for the appealing quinazolinone-1,2,3-triazole glycoside **13** was used to clearly show the levels of p53, Bax, Bcl-2, and in HCT-116 cells.

### Molecular docking simulation

Computational docking simulation has helped to facilitate the justification of biological discoveries. The auspicious quinazolinone-1,2,3-triazole glycosides **11** and **13** were docked into the active pockets of EGFR and VEGFR-2 (PDB codes: 1M17 and 4ASD, respectively)^62–64^ utilizing 2014.0901 edition among the MOE-Dock (Molecular Operating Environment) software^60,61^. Complete explanations can be found in the supporting documentation.

## Electronic supplementary material

Below is the link to the electronic supplementary material.

Supplementary Material 1

## Data Availability

The datasets used and/or analyzed during the current study available from the corresponding author on reasonable request.
