# Supplementary material for: Novel quinazolin-4-one based derivatives bearing 1,2,3-triazole and glycoside moieties as potential cytotoxic agents through dual EGFR and VEGFR-2 inhibitory activity
Source: Sci Rep. 2024 Oct 23;14:24980. doi: 10.1038/s41598-024-73171-8 (PMC11500008; doi:10.1038/s41598-024-73171-8)
Supplement: Supplementary file 1 — Supplementary Material 1 [file 41598_2024_73171_MOESM1_ESM.docx]

**Supplementary material**

**Novel Quinazolin-4-one Based Derivatives Bearing 1,2,3-Triazole and Glycoside Moieties as Potential Cytotoxic Agents through Dual EGFR and VEGFR-2 Inhibitory Activity**

Adel A.-H. Abdel-Rahman^a,*^, Mohamed N. El-Bayaa^b,c^, Asmaa Sobhy^a^, Eman M. El-Ganzoury^a^, Eman S. Nossier^d,e^, Hanem M. Awad^f^, Wael A. El-Sayed^b,c^

^a^Chemistry Department, Faculty of Science, Menoufia University, Shebin El-Kom, Egypt

^b^Department of Chemistry, College of Science, Qassim University, Buraidah 51452, Saudi Arabia.

^c^Photochemistry Department, National Research Centre, Dokki, P.O. Box 12622, Cairo, Egypt.

^d^Department of Pharmaceutical Medicinal Chemistry and Drug Design Department, Faculty of Pharmacy (Girls), Al-Azhar University, Cairo, 11754, Egypt.

^e^The National Committee of Drugs, Academy of Scientific Research and Technology, Cairo, 11516, Egypt.

^f^Tanning Materials and Leather Technology Department, National Research Centre, Dokki, Giza, 12622, Egypt.

**Correspondence:** Adel.Nassar@science.menofia.edu.eg

1. **Experimental**
   1. **Chemistry**

The utilized chemicals, solvents, and reagents were supplied from commercial suppliers and were of analytical grade (Sigma Aldrich, Fluka, Acros, BDH, or Merk), and employed without any additional purification. Infrared spectra were performed on a Nicolet FT-IR spectrophotometer in the range 4000-400 cm^-1^. The ^1^H NMR spectra were recorded in CDCl_3_-*D* and DMSO-*D*_6_ at 500 MHz using a Varian Gemini 200 NMR spectrometer. Fast Atom Bombardment (FAB) mass spectra for the ligands were carried out on a Shimadzu Qp-2010 Plus spectrometer. Melting points have been measured by utilizing the Stuart melting point apparatus. Analysis of the elements (C, H, and N) was performed on a Perkin Elmer-2400 elemental analyzer at micro analytical center of Cairo University.

**
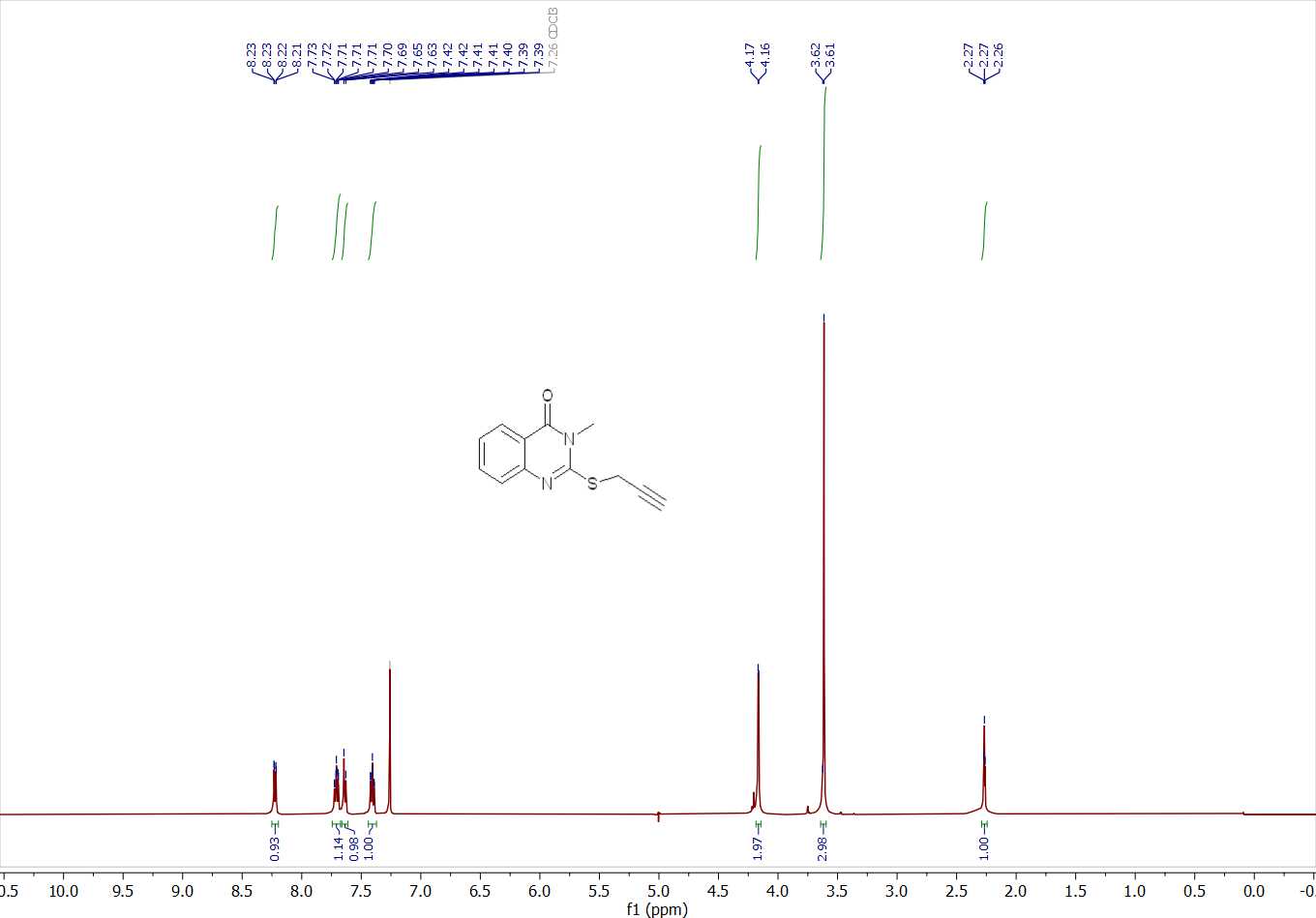
**

**Figure S1.** ^1^H NMR spectrum of compound **4** (500 MHz, CDCl_3_, 25 °C).

**
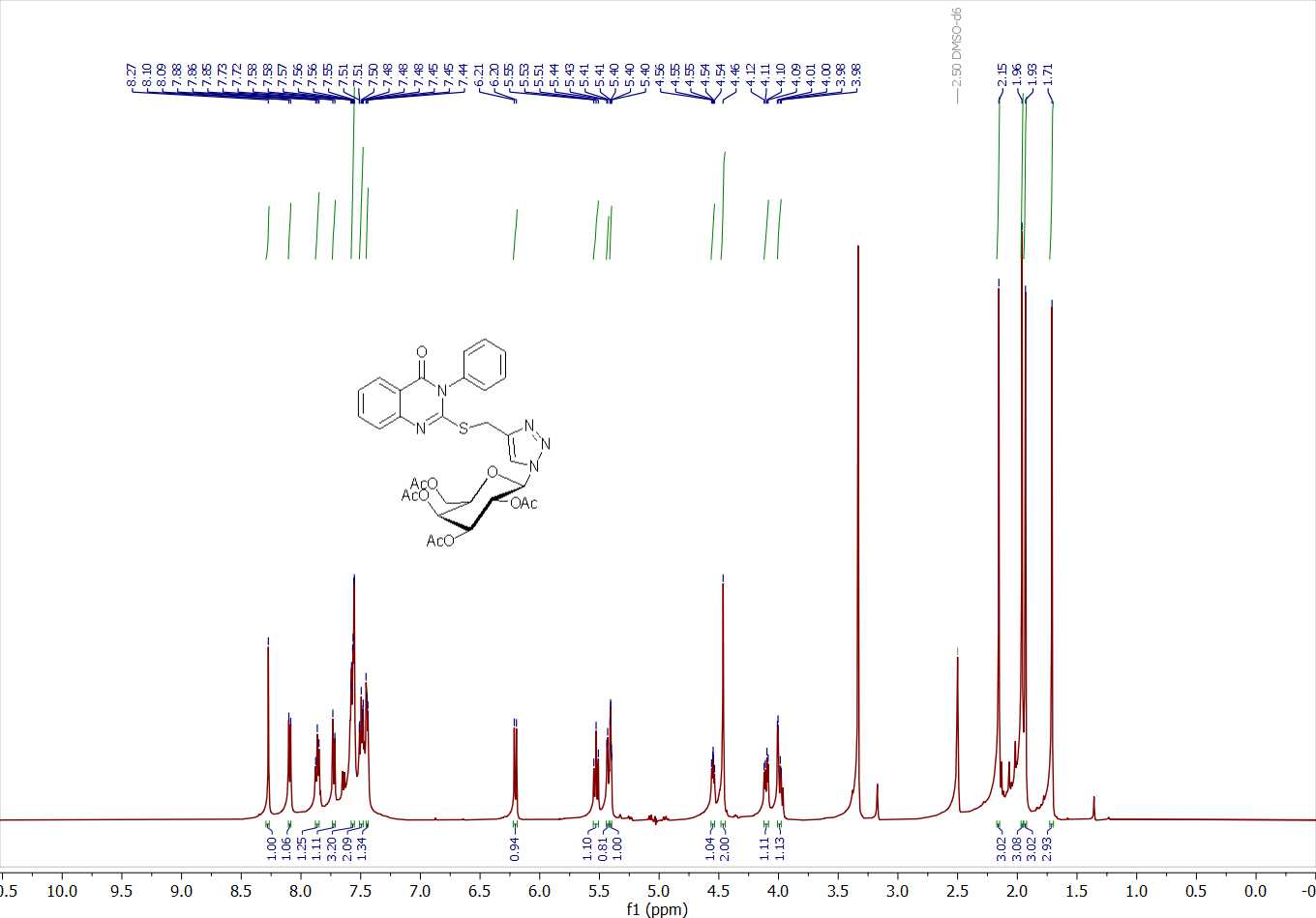
**

**Figure S2.** ^1^H NMR spectrum of compound **6** (500 MHz, DMSO, 25 °C).

**
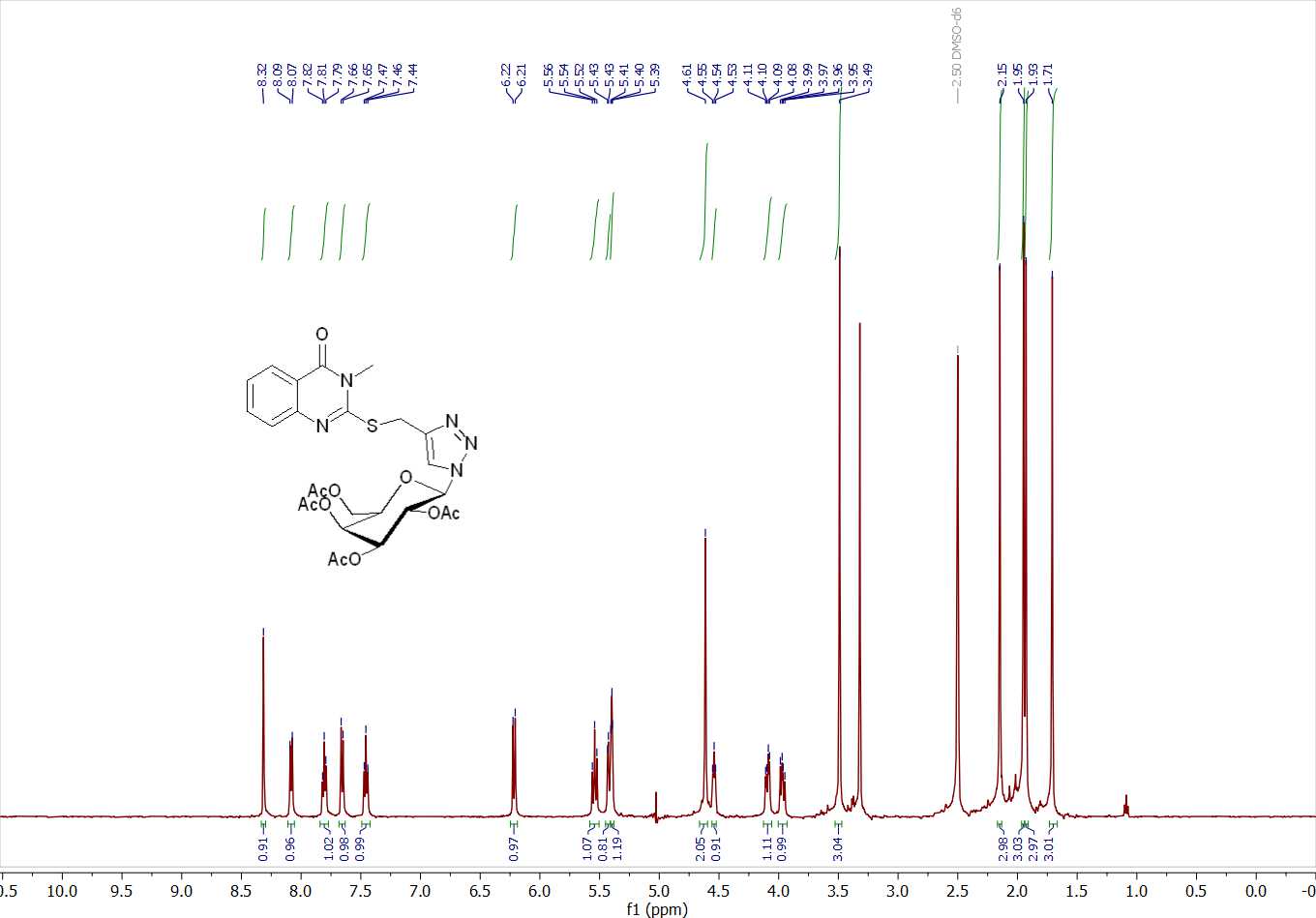
**

**Figure S3.** ^1^H NMR spectrum of compound **7** (500 MHz, DMSO, 25 °C).

**
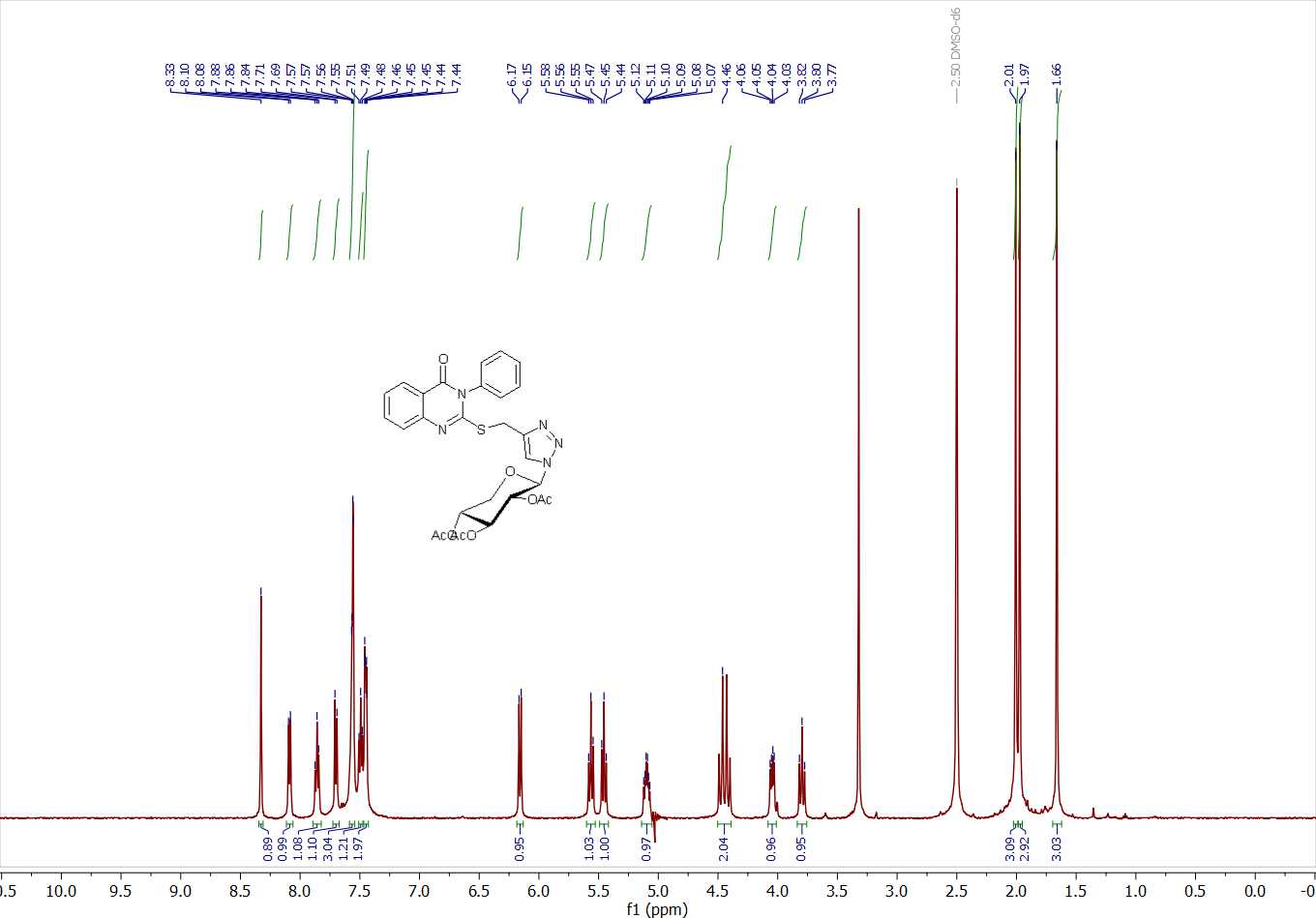
Figure S4.** ^1^H NMR spectrum of compound **8** (500 MHz, DMSO, 25 °C).

**
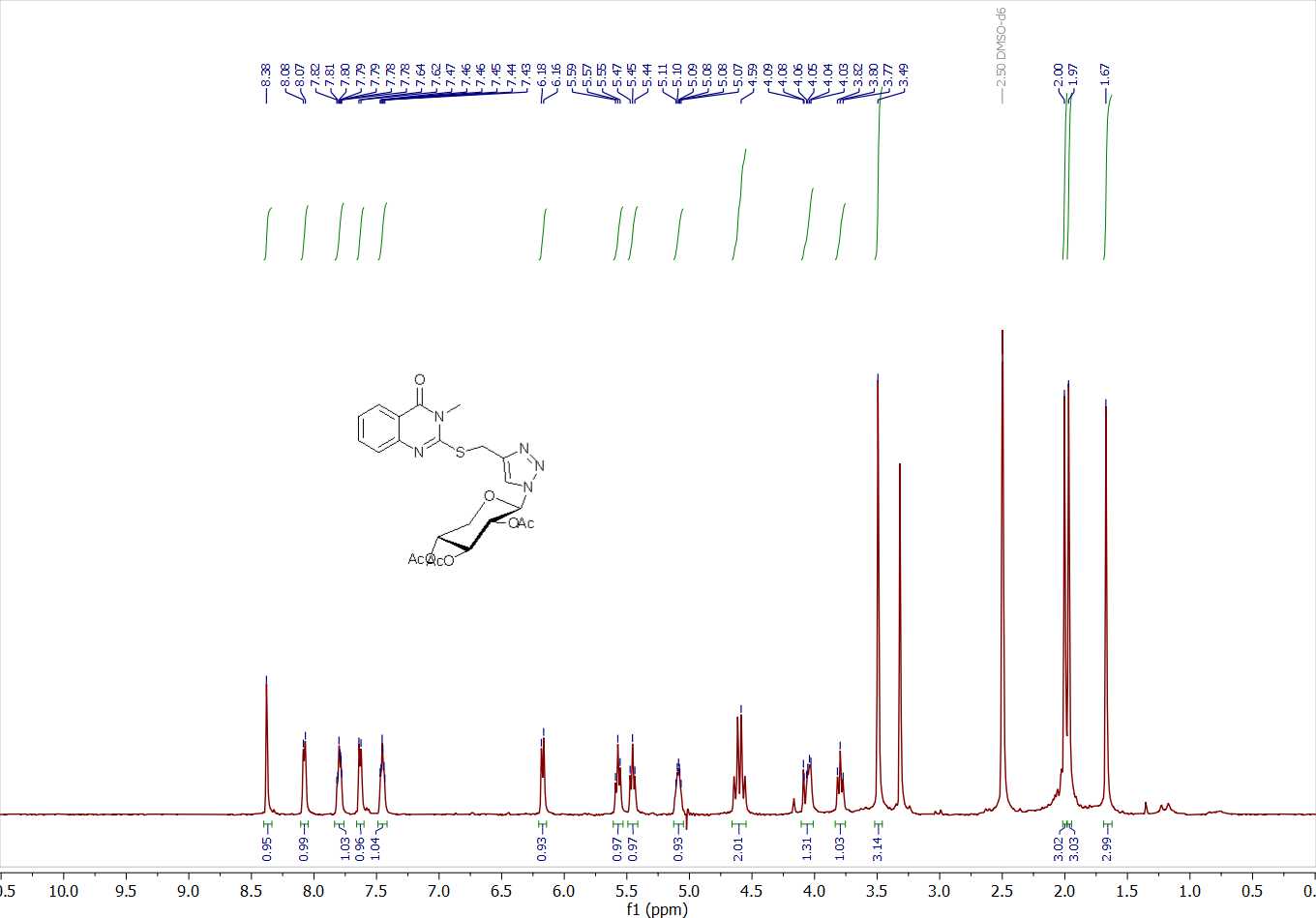
**

**Figure S5.** ^1^H NMR spectrum of compound **9** (500 MHz, DMSO, 25 °C).

**
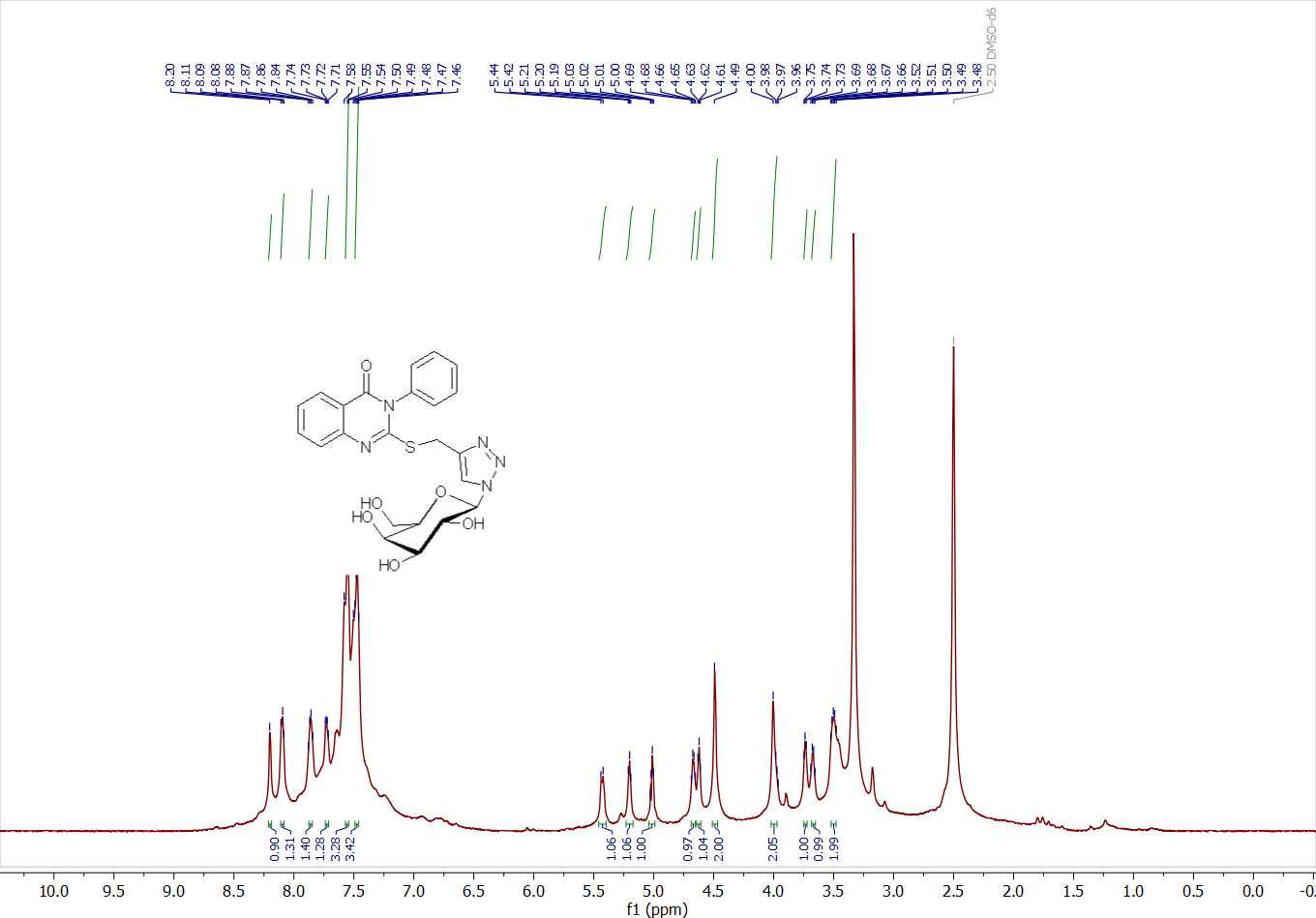
**

**Figure S6.** ^1^H NMR spectrum of compound **10** (500 MHz, DMSO, 25 °C).

**
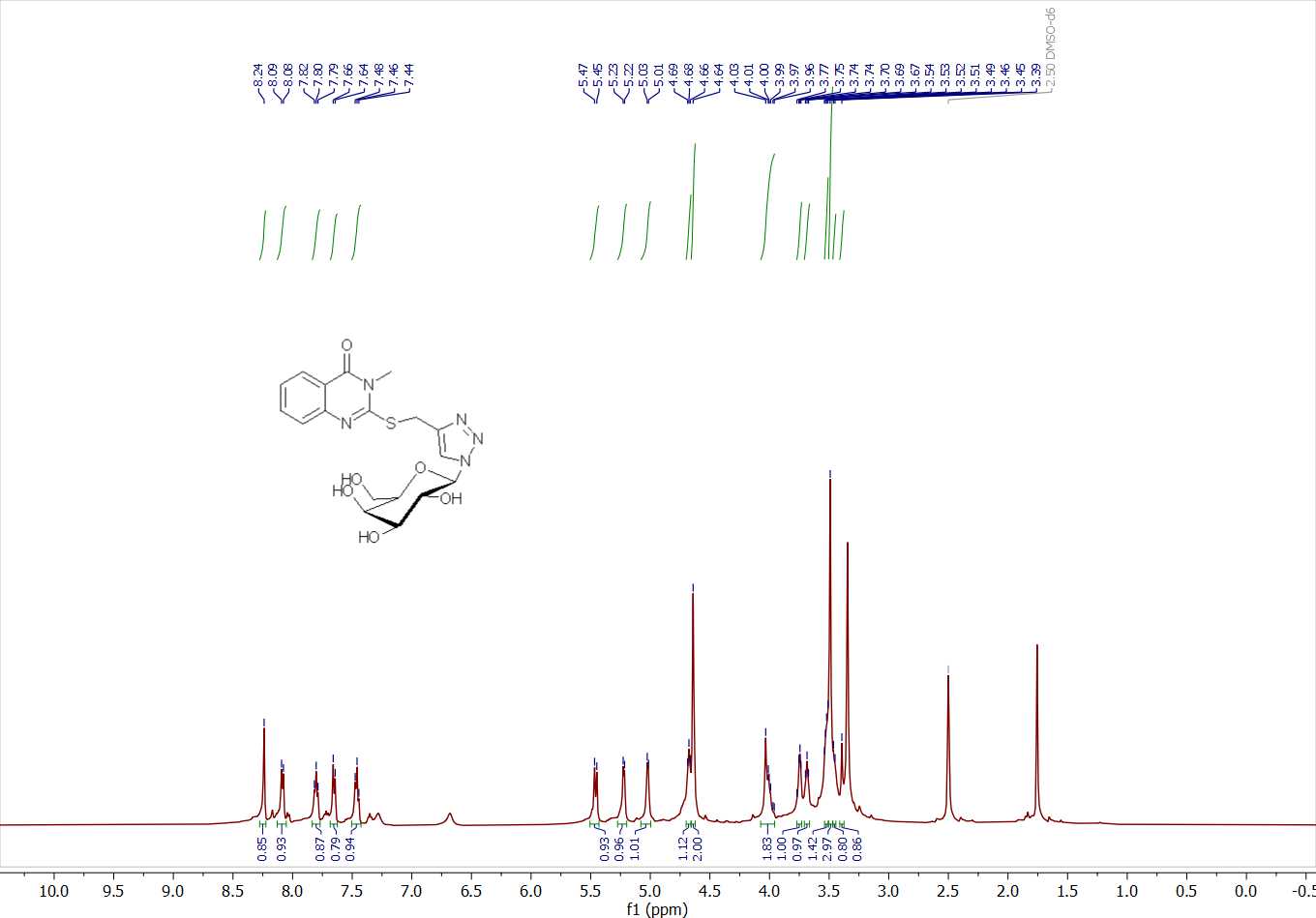
**

**Figure S7.** ^1^H NMR spectrum of compound **11** (500 MHz, DMSO, 25 °C).

**
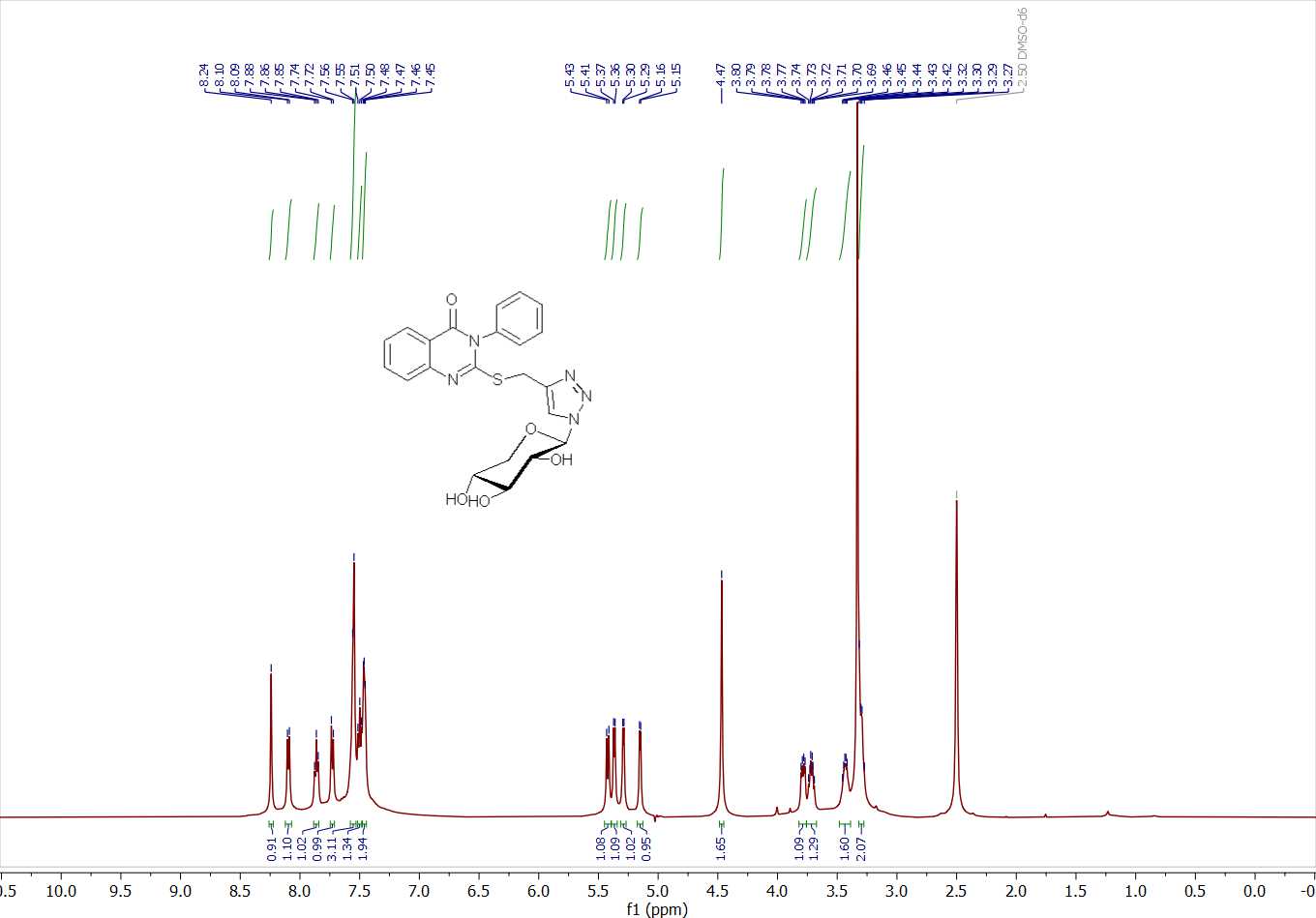
**

**Figure S8.** ^1^H NMR spectrum of compound **12** (500 MHz, DMSO, 25 °C).

**
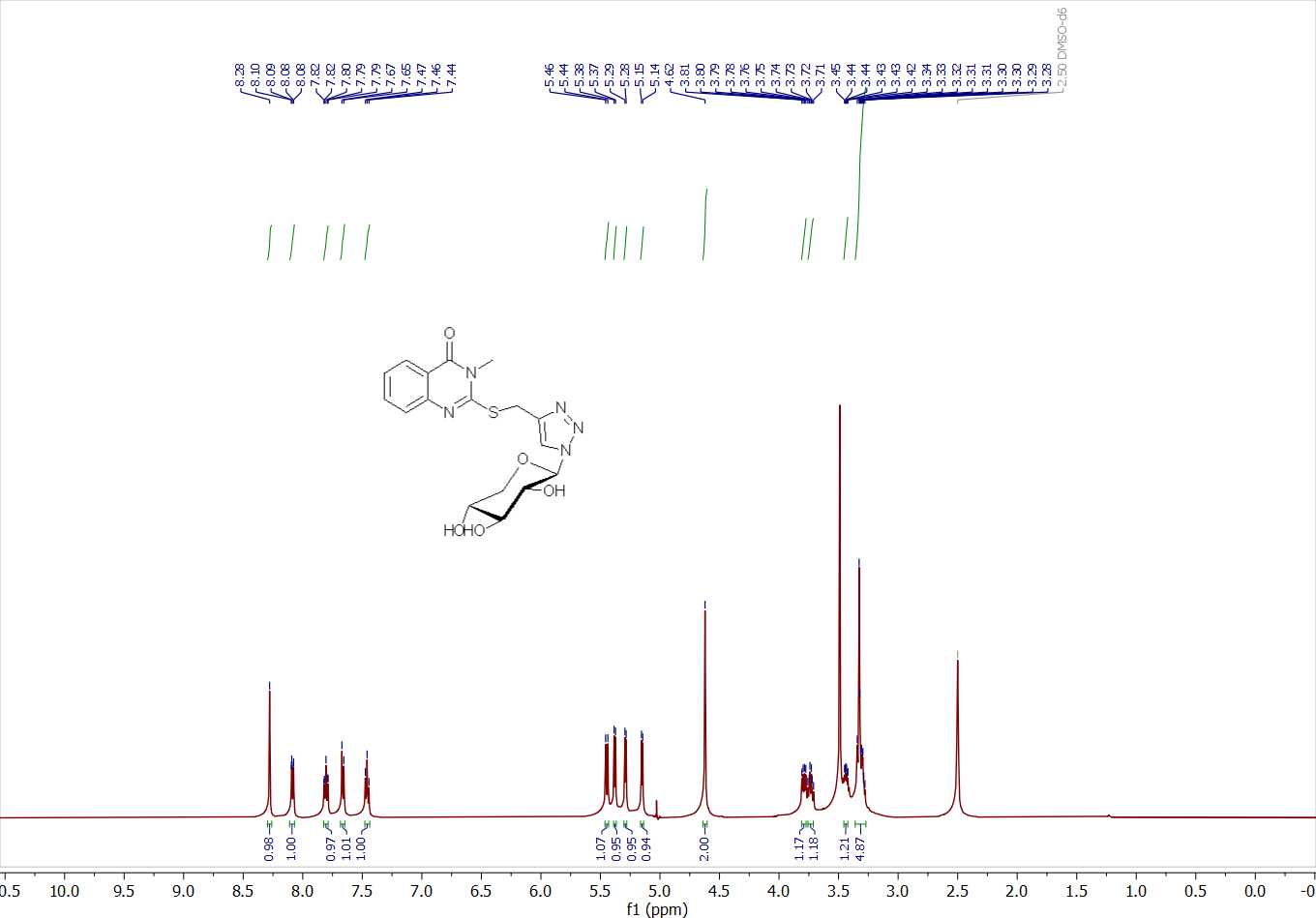
 Figure S9.** ^1^H NMR spectrum of compound **13** (500 MHz, DMSO, 25 °C).


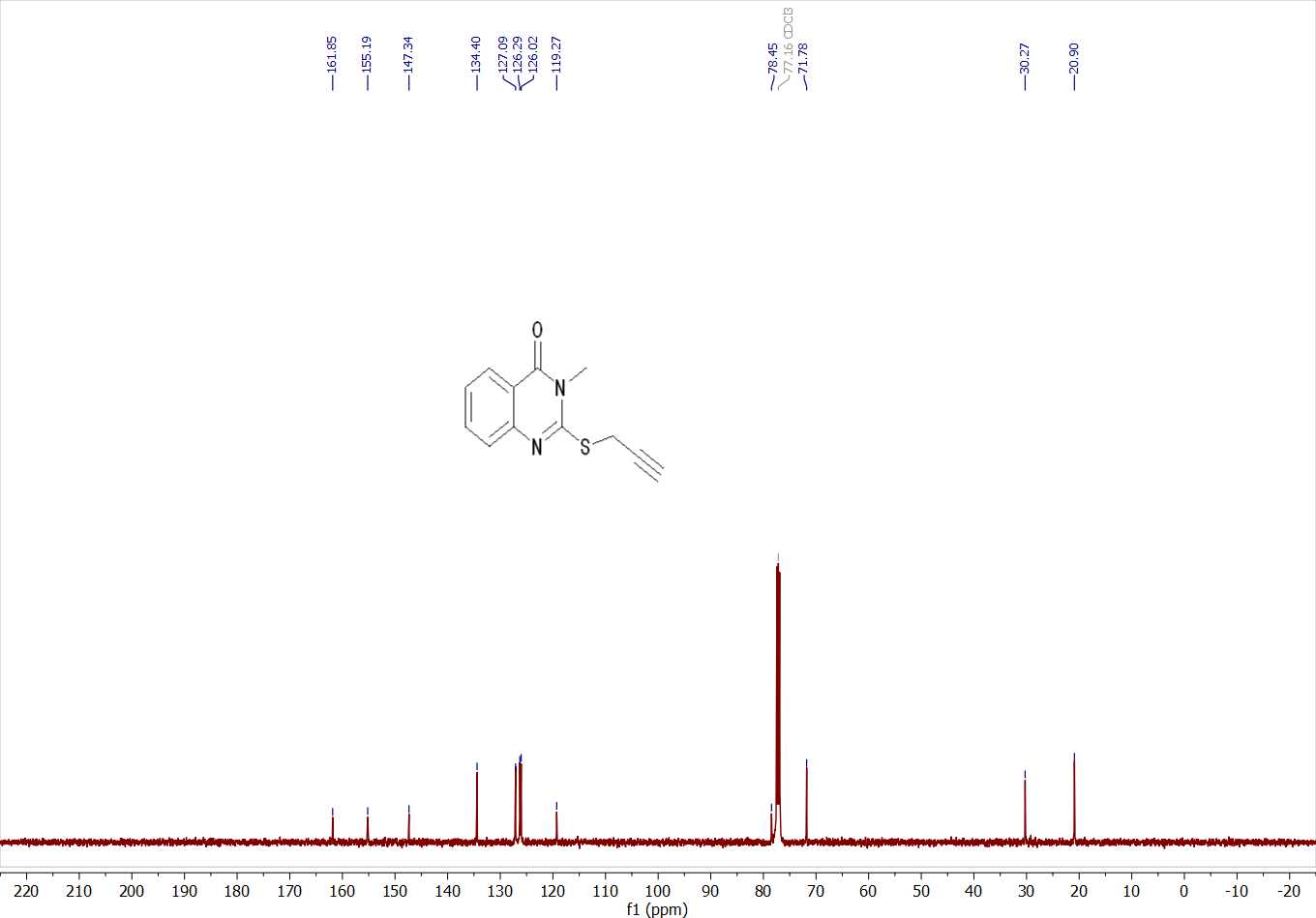


**Figure S10.** ^13^C NMR spectrum of compound **4** (500 MHz, CDCl_3_, 25 °C).

**
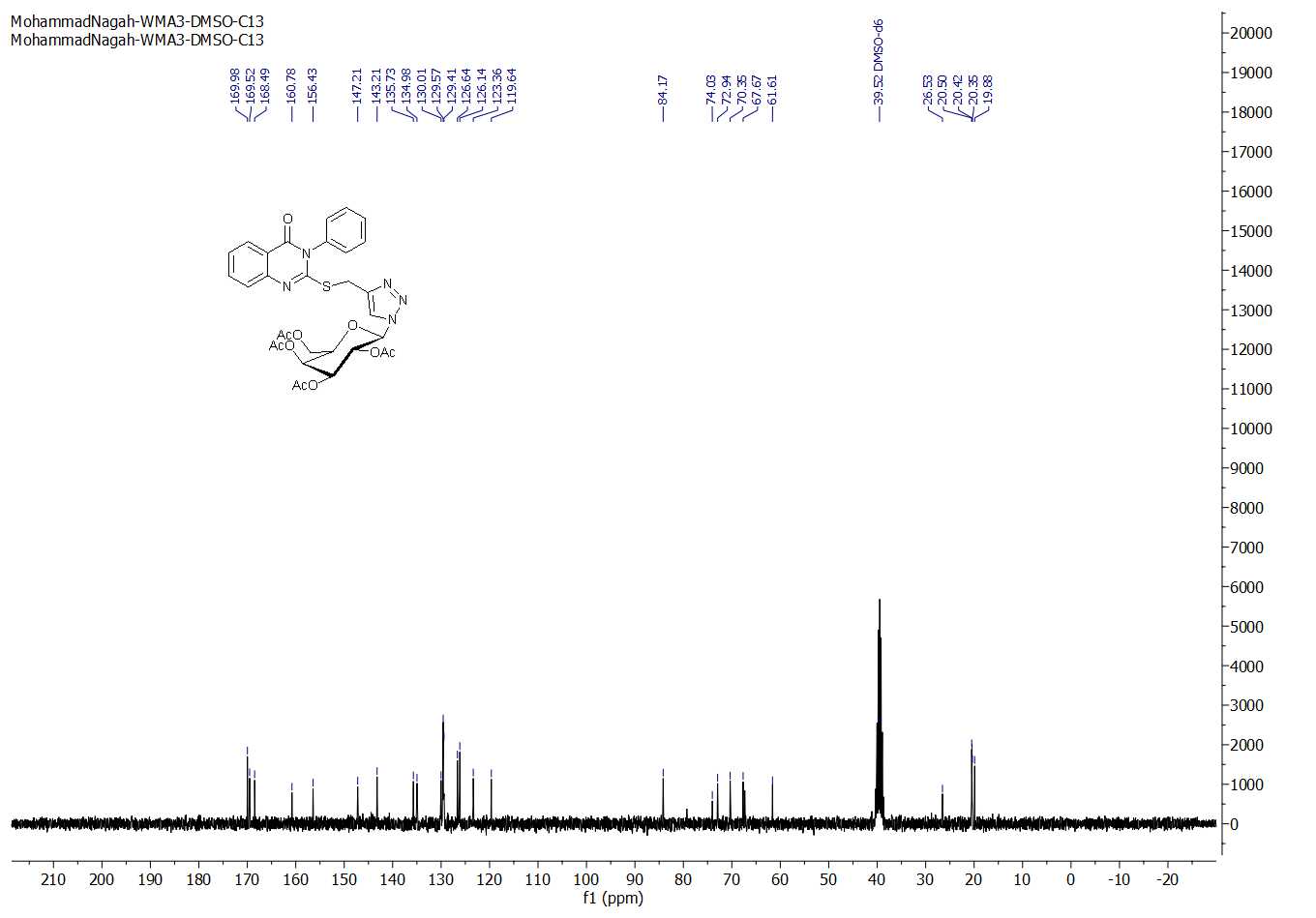
Figure S11.** ^13^C NMR spectrum of compound **6** (500 MHz, DMSO, 25 °C).

**
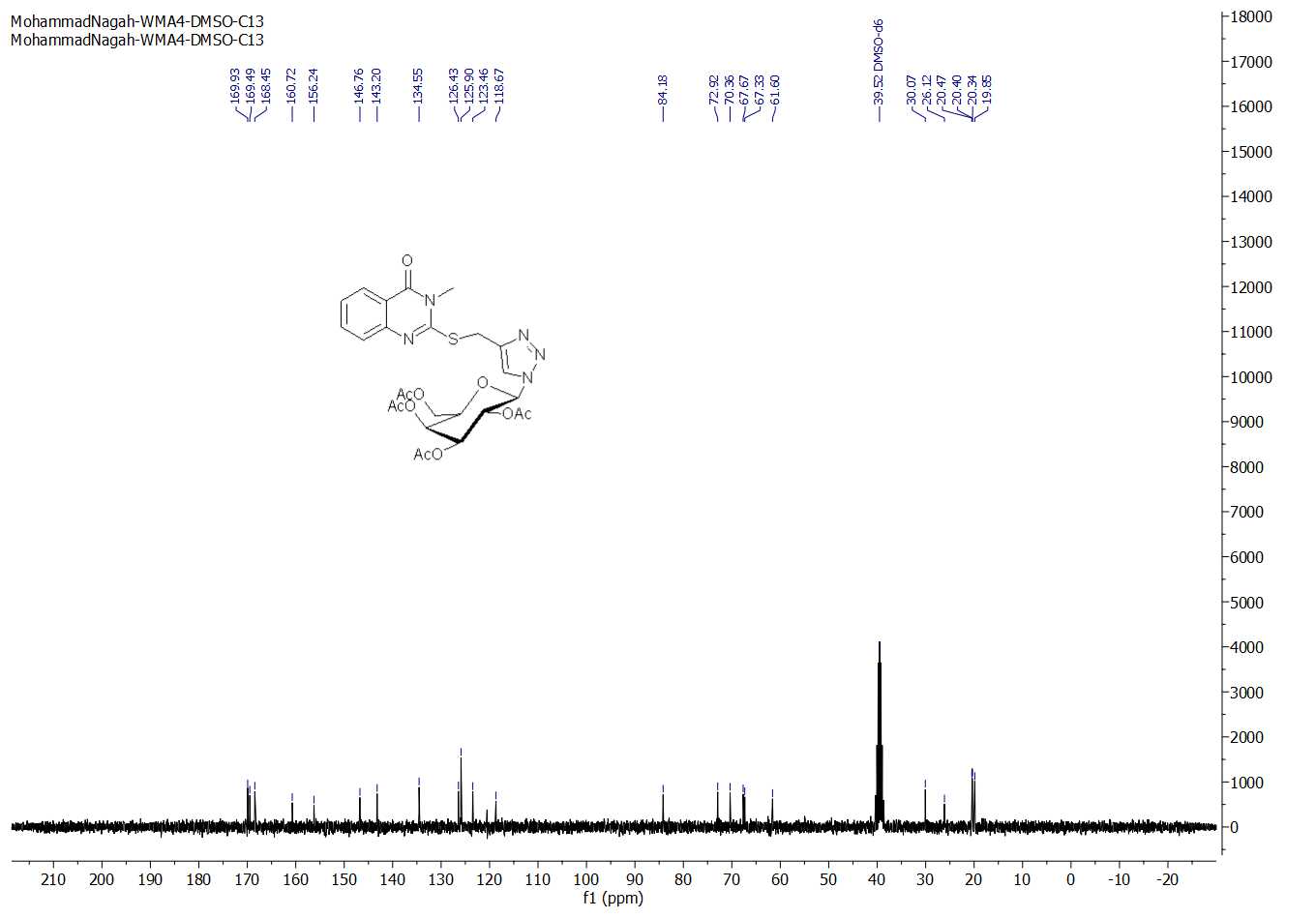
Figure S12.** ^13^C NMR spectrum of compound **7** (500 MHz, DMSO, 25 °C).

**
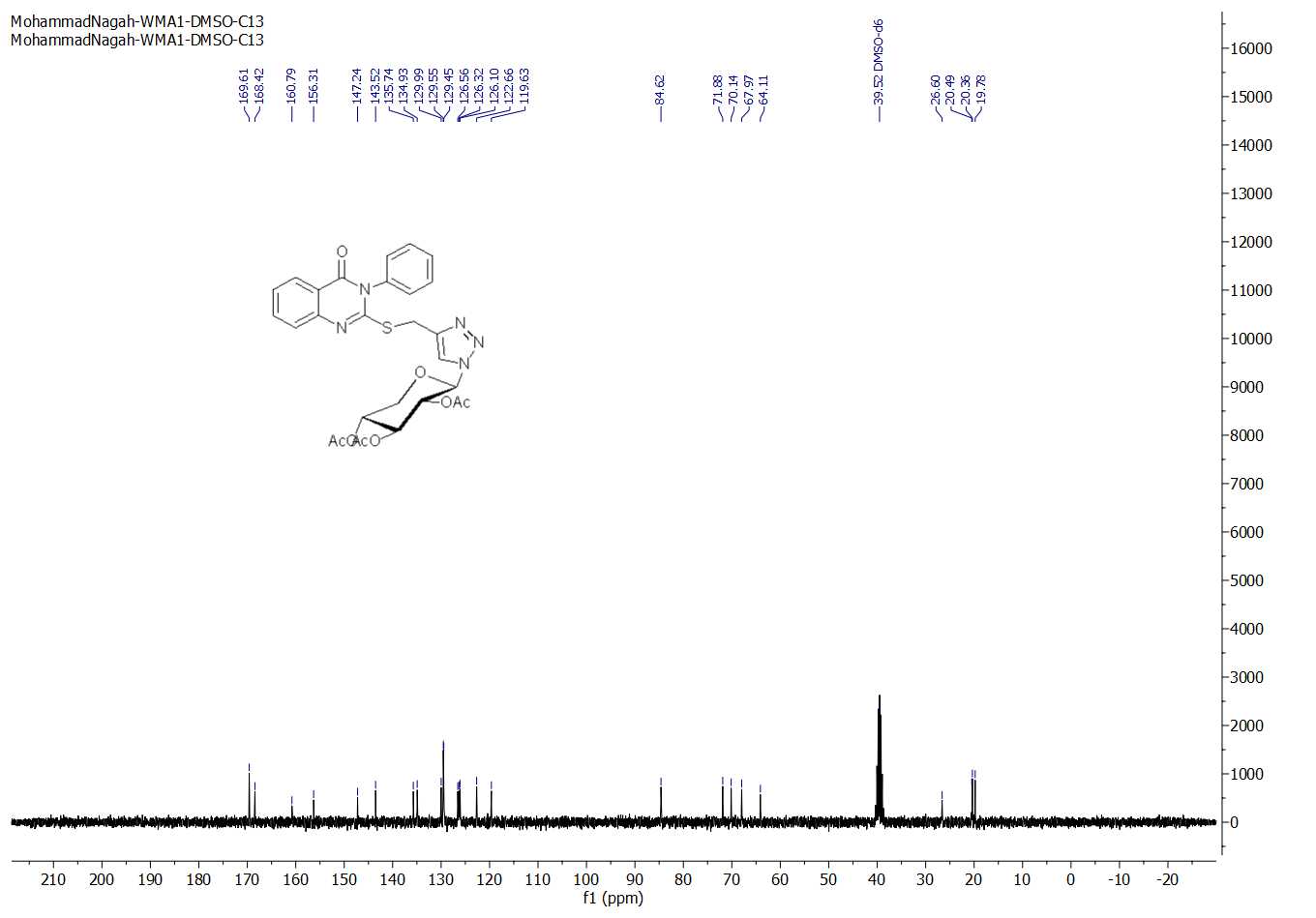
Figure S13.** ^13^C NMR spectrum of compound **8** (500 MHz, DMSO, 25 °C).

**
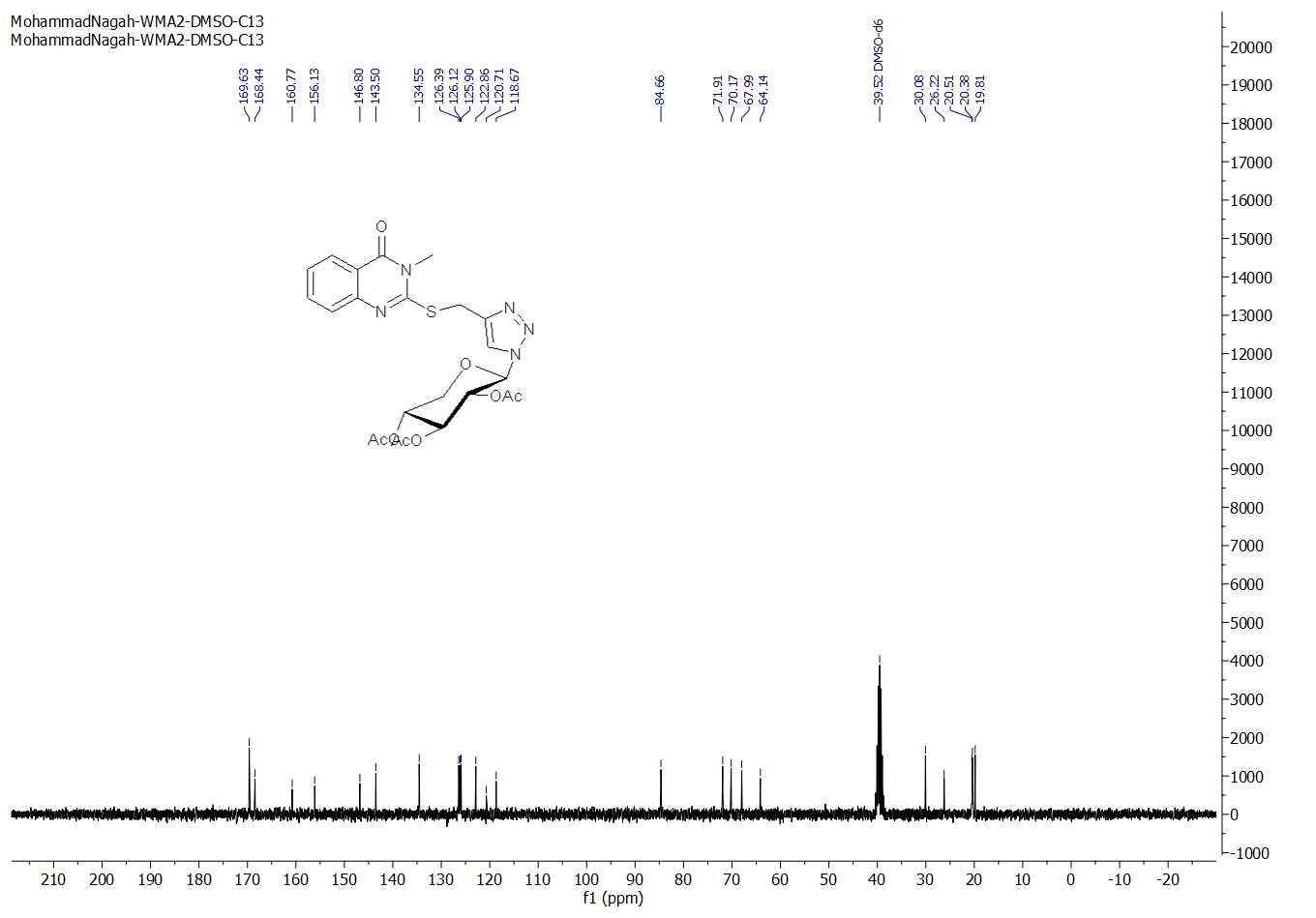
Figure S14.** ^13^C NMR spectrum of compound **9** (500 MHz, DMSO, 25 °C).

**
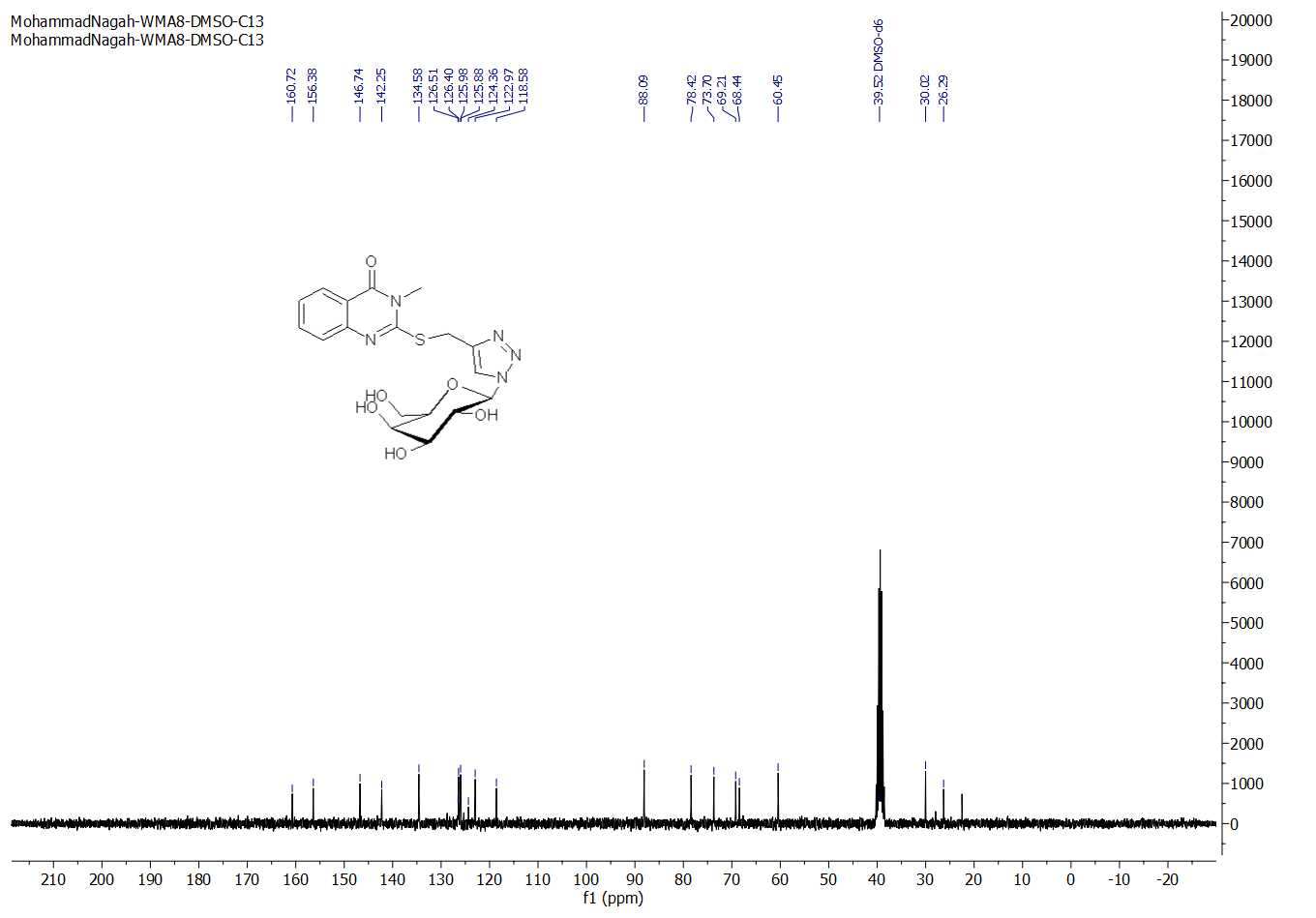
Figure S15.** ^13^C NMR spectrum of compound **11** (500 MHz, DMSO, 25 °C).

**
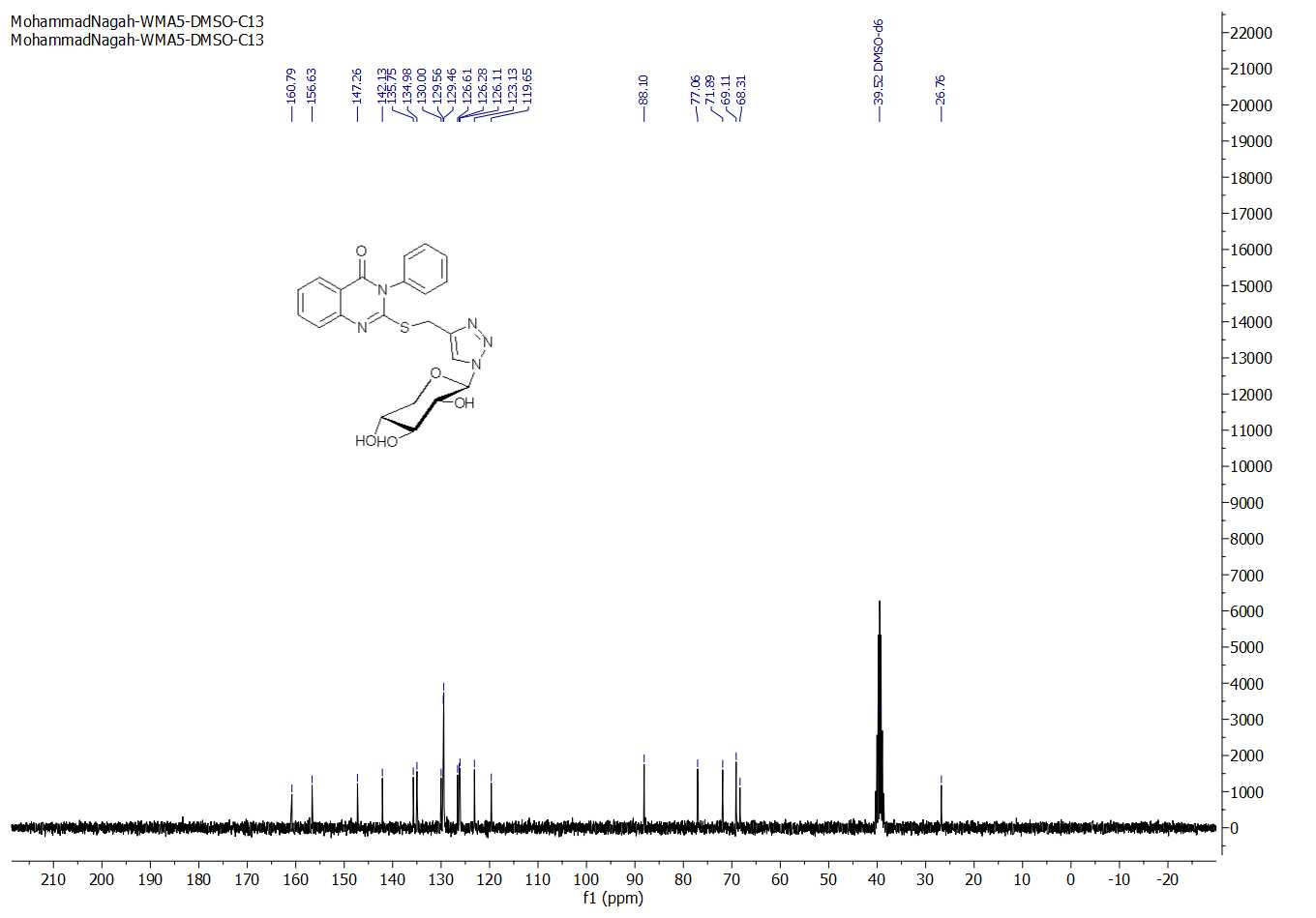
Figure S16.** ^13^C NMR spectrum of compound **12** (500 MHz, DMSO, 25 °C**
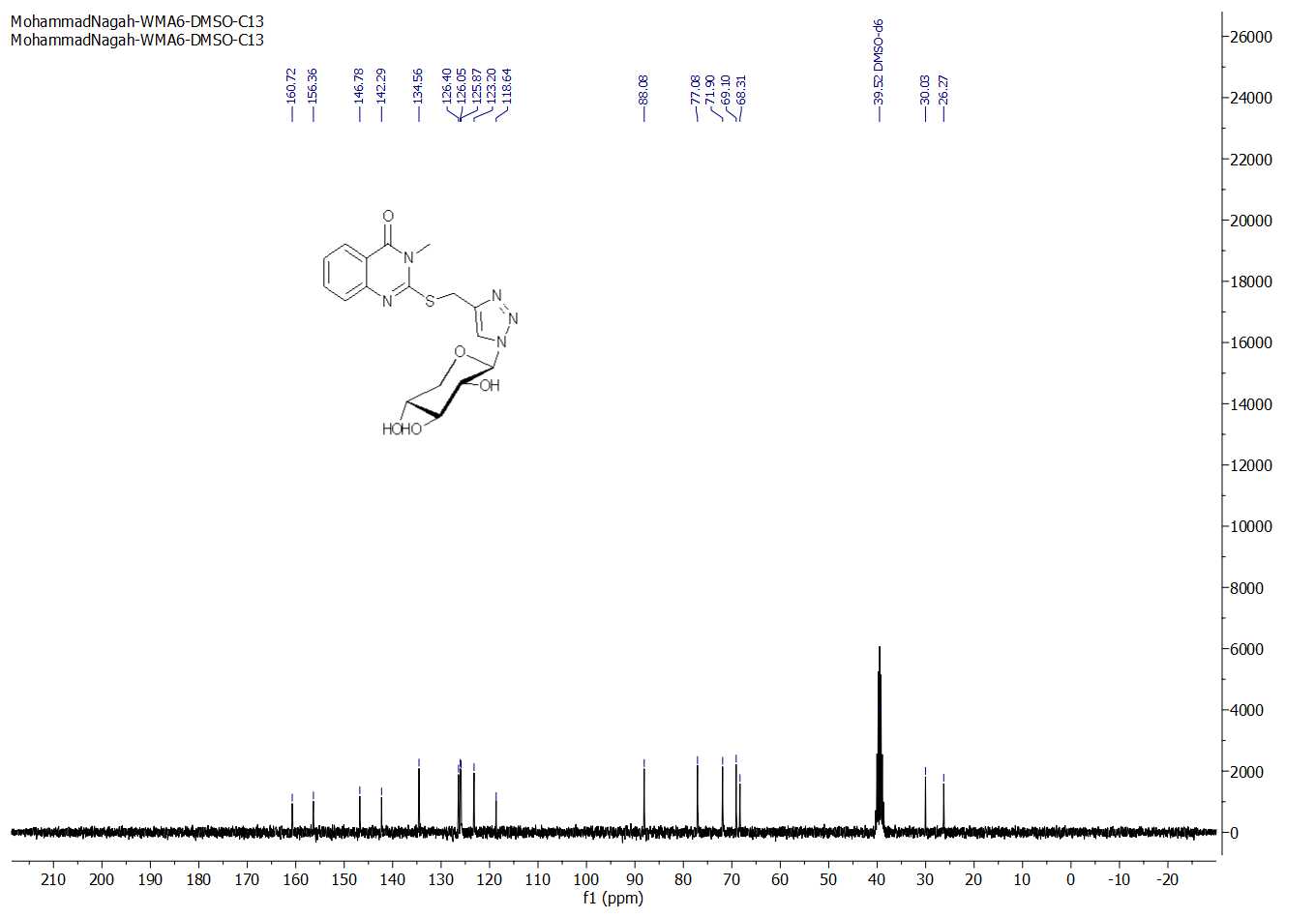
 Figure S17.** ^13^C NMR spectrum of compound **13** (500 MHz, DMSO, 25 °C).

- 1. **Biological evaluation**
     1. ***In vitro* cytotoxic screening**

The cell lines were obtained from Karolinska Center, Department of Oncology and Pathology, Karolinska Institute and Hospital, Stockholm, Sweden as follows: human liver HepG-2, breast MCF-7, and colorectal HCT-116 cancer cell lines and human fibroblast-derived BJ-1 normal cell line. Exponentially, cells were placed in 10^4^ cells/ well for 24 h, and then add fresh medium which containing different concentration of the tested sample. Serial two-fold dilution of the tested sample were added using a multichannel pipette. Moreover, all cells were cultivated at 37 °C, 5% CO_2_ and 95% humidity. Also, incubation of control cells occurred at 37 °C. However, after incubation for 24 h different concentrations of sample (25, 12.5, 6.25 and 3.125 µM) were added and continued the incubation for 48 h, then, add the crystal violet solution 1% to each well for 0.5 h to examine viable cells. Rinse the wells using water until no stain. After that, add 30% glacial acetic acid to all wells with shaking plates on Microplate reader (TECAN, Inc.) to measure the absorbance, using a test wavelength of 490 nm. Besides, compare the treated samples with the control cell. The cytotoxicity was estimated by IC_50_ in (μM), the concentration that inhibits 50% of growth of cancer cell.

- - 1. ***In vitro* inhibition assay of EGFR and VEGFR-2 activities**

EGFR assay: The master mixture (6 μL 5X Kinase Buffer + 1 μL ATP (500 μM) + 1 μL 50 X PTK substrate + 17 μL water) was prepared then, 25 μL to every well was added. 5 μL of Inhibitor solution of each well labeled as “Test Inhibitor” was added. However, for the “Positive Control" and “Blank”, 5 μL of the same solution without inhibitor (Inhibitor buffer) was added. 3 mL of 1X Kinase Buffer by mixing 600 μL of 5X Kinase Buffer with 2400 μL water was prepared. So, 3 mL of 1X Kinase Buffer became sufficient for 100 reactions. To the wells designated as "Blank", 20 μl of 1X Kinase Buffer was added. EGFR enzyme on ice was thawed. Upon first thaw, briefly the tube containing enzyme was spun to recover full content of the tube. The amount of EGFR required for the assay and dilute enzyme to 1 ng/μL with 1X Kinase Buffer was calculated. Moreover, the remaining undiluted enzyme in aliquots was stored at –80°C. The reaction was initiated by adding 20 μL of diluted EGFR enzyme to the wells designated “Positive Control” and "Test Inhibitor Control", after that it was incubated at 30°C for 40 minutes. After the 40 minutes reaction, 50 μL of Kinase-Glo Max reagent was added to each well and the plate was covered with aluminum foil and incubated at room temperature for 15 min. Luminescence was measured using the microplate reader.

VEGFR-2 assay: the effect of the most promising cytotoxic quinazolinone-based derivatives **6**–**13** on the level of VEGFR-2 in human colorectal cancer cell lines HCT-116 was determined. The cells in culture medium were treated with 20 μl of IC_50_ values of the compounds dissolved in DMSO, then incubated for 24 hours at 37 ºC, in a humidified 5% CO_2_ atmosphere. The cells were harvested and the homogenates were prepared in saline using a tight pestle homogenizer until complete cell disruption. The kit uses a double-antibody sandwich enzyme-linked immunosorbent assay (ELISA) to determine the level of human VEGFR-2 in samples. A monoclonal antibody for VEGFR-2 was pre-coated onto 96-well plates. The test samples are added to the wells and a biotinylated detection polyclonal antibody from goat specific for VEGFR-2 was added subsequently followed by washing with PBS buffer. Avidin-Biotin-Peroxidase complex was added and the unbound conjugates were washed away with PBS buffer. HRP substrate TMB was used to visualize HRP enzymatic reaction. TMB was catalyzed by HRP to produce a blue color product that changed into yellow after adding acidic stop solution. The density of yellow color is proportional to the human VEGFR-2 amount of the sample captured in the plate. The chroma of color and the concentration of the human VEGFR-2 of the samples were positively correlated and the optical density was determined at 450 nm. The level of human VEGFR-2 in samples was calculated (pg/ml) as duplicate determinations from the standard curve. Percent inhibition was calculated in comparison to control untreated cells.

- - 1. **Cell cycle arrest and apoptosis of compound 13**

Cell cycle analysis and apoptosis study were carried out using flow cytometry. HCT-116 cells were seeded at 8×10^4^ and incubated at 37°C in 5% CO_2_ overnight. After treatment with the tested compound 10 for 24 h, cell pellets were collected and centrifuged (300 g, 5 min). For cell cycle analysis cell pellets were fixed with 70% ethanol on ice for 15 min and collected again. The pellets were incubated with propidium iodide (PI) staining solution at room temperature for 1 h and analyzed by a Gallios flow cytometer (Beckman Coulter, Brea, CA, USA). Apoptosis detection was carried out by FITC AnnexinV/PI commercial kit (Becton Dickenson, Franklin Lakes, NJ, USA) following the manufacturer protocol. The samples were analyzed by fluorescence-activated cell sorting (FACS) with a Gallios flow cytometer (Beckman Coulter, Brea, CA, USA) within 1 h after staining. Data were analyzed using Kaluza v 1.2 (Beckman Coulter).

- - 1. **Estimation the levels of p53, Bax and Bcl-2.**

The levels of the apoptotic marker Bax and anti-apoptotic marker Bcl-2 were estimated using BIO RAD iScript TM One-Step RT-PCR kit with SYBR® Green. The procedure of the used kit was done according to the manufacturer’s instructions.

Human p53 present in HCT-116 cells was determined; using Human p53 ELISA-Kit (CS0070 Sigma) read using spectrophotometer at 450 nm against untreated control cells (negative control) applying the standard protocols of the manufacturers. The samples or standard having human p53 bind to antibodies adsorbed to the microwells. Addition of biotin-conjugated was followed by incubation and addition of dispense of unbound biotin-conjugated streptavidin HRP. Then, the reaction was terminated by adding acid, and the absorbance was measured at 450 nm.

- 1. **Molecular docking study**

The molecular docking simulation of the promising *in vitro* screened quinazolinone-1,2,3-triazole glycosides **11** and **13** against EGFR and VEGFR-2 was done using the Molecular Operating Environment software (MOE-Dock) version 2014.0901. The co-crystallized structures of EGFR and VEGFR-2 complexed with their native ligands, erlotinib and sorafenib were downloaded from the protein data bank (PDB codes: 1M17 and 4ASD, respectively). All minimizations were performed using MOE until an RMSD gradient of 0.05 kcal∙mol^−1^Å^−1^ with MMFF94x force field and the partial charges were automatically calculated. Preparation of the enzyme structures was done for molecular docking using Protonate 3D protocol with the default options in MOE. London dG scoring function and Triangle Matcher placement method were used in the docking protocol. Initially, the original ligands were re-docked into the active binding sites of EGFR and VEGFR-2 to assess the root-mean-square deviation values. Then, the docking studies of the newly targeted compounds were estimated within the ATP-binding sites after elimination of the co-crystallized ligands.

**Tables and figures**

**Table S1**. The antitumor activities of the synthesized quinazolin-4-ones **1**–**4, 6**–**13** against cancerous HepG-2, MCF-7, HCT-116 and normal BJ-1 cell lines comparing with doxorubicin expressed as IC_50_ values.

| **IC_50_ (mean±SD) (µM)** | | | | **Compd. No.** |
| --- | --- | --- | --- | --- |
| **BJ-1** | **HCT-116** | **MCF-7** | **HepG-2** |  |
| 56.1 ± 4.3 | 18.6 ± 1.5 | 16.1 ± 1.1 | 21.9 ± 1.9 | **1** |
| 65.8 ± 5.5 | 16.1 ± 1.3 | 14.9 ± 1.3 | 24.1 ± 3.1 | **2** |
| 73.9 ± 5.5 | 14.8 ± 1.6 | 15.9 ± 1.3 | 25.7 ± 2.5 | **3** |
| 59.5 ± 4.1 | 13.5 ± 1.1 | 14.6 ± 1.1 | 21.9 ± 2.7 | **4** |
| 38.1 ± 3.7 | 3.2 ± 0.1 | 13.4 ± 1.2 | 19.2 ± 2.1 | **6** |
| 22.9 ± 3.2 | 5.5 ± 0.1 | 17.3 ± 0.5 | 21.5± 2.5 | **7** |
| 30.0 ± 3.4 | 2.9 ± 0.1 | 14.0 ± 1.2 | 21.4 ± 3.1 | **8** |
| 67.5 ± 4.7 | 3.2 ± 0.2 | 12.6 ± 1.3 | 22.9 ± 2.3 | **9** |
| 23.5 ± 3.1 | 6.4 ± 0.4 | 8.1 ± 0.6 | 17.1 ± 1.1 | **10** |
| 34.4 ± 2.9 | 5.9 ± 0.1 | 5.7 ± 0.2 | 17.8 ± 2.1 | **11** |
| 24.7 ± 2.4 | 3.0 ± 0.3 | 6.9 ± 0.3 | 15.1 ± 1.9 | **12** |
| 23.5 ± 2.3 | 5.4 ± 0.3 | 7.9 ± 0.3 | 18.2 ± 2.2 | **13** |
| 32.1± 3.1 | 6.5 ± 0.5 | 5.6 ± 0.3 | 4.8 ± 0.5 | **Doxorubicin** |
| 58.3± 1.4 | 7.3 ±0.2 | 4.3±0.1 | 7.8±0.2 | **Erlotinib** |
| IC_50_: Compound concentration required to inhibit growth by 50%, SD: Standard deviation; each value is the mean of three values, (–) not detected. | | | | |

.

**Figure S18.** Dose dependent antiproliferative activity upon human HepG-2 tumor cell lines at different concentrations.

**Figure S19.** Dose dependent antiproliferative activity upon human MCF-7 tumor cell lines at different concentrations.

**Figure S20.** Dose dependent antiproliferative activity upon human HCT-116 tumor cell lines at different concentrations.

**Figure S21.** Dose dependent antiproliferative activity upon human normal fibroblast-derived BJ-l cell lines at different concentrations.

**Table S2.** Cell cycle analysis after 48 h incubation with compound **13**

| **Compd. No.** | **%G0-G1** | **%S** | **%G2/M** |
| --- | --- | --- | --- |
| **13** / **HCT-116** | 68.11 | 22.46 | 9.43 |
| **cont./** **HCT-116** | 53.95 | 32.81 | 13.24 |

**Table S3.** Apoptosis induction analysis within HCT-116 cells treated with compound **13**

|  | Apoptosis | | | | Necrosis |
| --- | --- | --- | --- | --- | --- |
|  | **Total** | | **Early** | **Late** |  |
| 13/ HCT-116 | | 36.31 | 7.42 | 25.36 | 3.53 |
| Cont. / HCT-116 | | 2.04 | 0.55 | 0.14 | 1.35 |

**Table S4.** Anticipated ADMET profile of the quinazolinone-1,2,3-triazole glycosides **10**–**13.**

| **Properties** | **Compound** | | | | |  |
| --- | --- | --- | --- | --- | --- | --- |
|  | **10** | **11** | **12** | | **13** | |
| **Absorption** | | | | | | |
| [BBB](http://lmmd.ecust.edu.cn/admetsar1/predict/?smiles=OC1%3DCC%3DC%28%5CC%3DN%5CNC2%3DN%5CC%28%3DC%2FC%3DC%2FC3%3DCC%3DCC%3DC3%29C%28%3DO%29N2%29C%3DC1+&action=A) | – | – | | – | – | |
| [HIA](http://lmmd.ecust.edu.cn/admetsar1/predict/?smiles=OC1%3DCC%3DC%28%5CC%3DN%5CNC2%3DN%5CC%28%3DC%2FC%3DC%2FC3%3DCC%3DCC%3DC3%29C%28%3DO%29N2%29C%3DC1+&action=A) | + (low) | +(low) | | +(low) | +(low) | |
| P-glycoprotein Substrate | [Non-substrate](http://lmmd.ecust.edu.cn/admetsar1/predict/?smiles=OC1%3DCC%3DC%28%5CC%3DN%5CNC2%3DN%5CC%28%3DC%2FC%3DC%2FC3%3DCC%3DCC%3DC3%29C%28%3DO%29N2%29C%3DC1+&action=A) | [Non-substrate](http://lmmd.ecust.edu.cn/admetsar1/predict/?smiles=OC1%3DCC%3DC%28%5CC%3DN%5CNC2%3DN%5CC%28%3DC%2FC%3DC%2FC3%3DCC%3DCC%3DC3%29C%28%3DO%29N2%29C%3DC1+&action=A) | | [Non-substrate](http://lmmd.ecust.edu.cn/admetsar1/predict/?smiles=OC1%3DCC%3DC%28%5CC%3DN%5CNC2%3DN%5CC%28%3DC%2FC%3DC%2FC3%3DCC%3DCC%3DC3%29C%28%3DO%29N2%29C%3DC1+&action=A) | [Non-substrate](http://lmmd.ecust.edu.cn/admetsar1/predict/?smiles=OC1%3DCC%3DC%28%5CC%3DN%5CNC2%3DN%5CC%28%3DC%2FC%3DC%2FC3%3DCC%3DCC%3DC3%29C%28%3DO%29N2%29C%3DC1+&action=A) | |
| P-glycoprotein Inhibitor | [Non-inhibitor](http://lmmd.ecust.edu.cn/admetsar1/predict/?smiles=OC1%3DCC%3DC%28%5CC%3DN%5CNC2%3DN%5CC%28%3DC%2FC%3DC%2FC3%3DCC%3DCC%3DC3%29C%28%3DO%29N2%29C%3DC1+&action=A) | [Non-inhibitor](http://lmmd.ecust.edu.cn/admetsar1/predict/?smiles=OC1%3DCC%3DC%28%5CC%3DN%5CNC2%3DN%5CC%28%3DC%2FC%3DC%2FC3%3DCC%3DCC%3DC3%29C%28%3DO%29N2%29C%3DC1+&action=A) | | [Non-inhibitor](http://lmmd.ecust.edu.cn/admetsar1/predict/?smiles=OC1%3DCC%3DC%28%5CC%3DN%5CNC2%3DN%5CC%28%3DC%2FC%3DC%2FC3%3DCC%3DCC%3DC3%29C%28%3DO%29N2%29C%3DC1+&action=A) | [Non-inhibitor](http://lmmd.ecust.edu.cn/admetsar1/predict/?smiles=OC1%3DCC%3DC%28%5CC%3DN%5CNC2%3DN%5CC%28%3DC%2FC%3DC%2FC3%3DCC%3DCC%3DC3%29C%28%3DO%29N2%29C%3DC1+&action=A) | |
| **Distribution** | | | | | | |
| Subcellular localization | [Mitochondria](http://lmmd.ecust.edu.cn/admetsar1/predict/?smiles=OC1%3DCC%3DC%28%5CC%3DN%5CNC2%3DN%5CC%28%3DC%2FC%3DC%2FC3%3DCC%3DCC%3DC3%29C%28%3DO%29N2%29C%3DC1+&action=A) | [Mitochondria](http://lmmd.ecust.edu.cn/admetsar1/predict/?smiles=OC1%3DCC%3DC%28%5CC%3DN%5CNC2%3DN%5CC%28%3DC%2FC%3DC%2FC3%3DCC%3DCC%3DC3%29C%28%3DO%29N2%29C%3DC1+&action=A) | | [Mitochondria](http://lmmd.ecust.edu.cn/admetsar1/predict/?smiles=OC1%3DCC%3DC%28%5CC%3DN%5CNC2%3DN%5CC%28%3DC%2FC%3DC%2FC3%3DCC%3DCC%3DC3%29C%28%3DO%29N2%29C%3DC1+&action=A) | [Mitochondria](http://lmmd.ecust.edu.cn/admetsar1/predict/?smiles=OC1%3DCC%3DC%28%5CC%3DN%5CNC2%3DN%5CC%28%3DC%2FC%3DC%2FC3%3DCC%3DCC%3DC3%29C%28%3DO%29N2%29C%3DC1+&action=A) | |
| **Metabolism** | | | | | | |
| CYP450 2C9 Substrate | [Non-substrate](http://lmmd.ecust.edu.cn/admetsar1/predict/?smiles=OC1%3DCC%3DC%28%5CC%3DN%5CNC2%3DN%5CC%28%3DC%2FC%3DC%2FC3%3DCC%3DCC%3DC3%29C%28%3DO%29N2%29C%3DC1+&action=A) | [Non-substrate](http://lmmd.ecust.edu.cn/admetsar1/predict/?smiles=OC1%3DCC%3DC%28%5CC%3DN%5CNC2%3DN%5CC%28%3DC%2FC%3DC%2FC3%3DCC%3DCC%3DC3%29C%28%3DO%29N2%29C%3DC1+&action=A) | | [Non-substrate](http://lmmd.ecust.edu.cn/admetsar1/predict/?smiles=OC1%3DCC%3DC%28%5CC%3DN%5CNC2%3DN%5CC%28%3DC%2FC%3DC%2FC3%3DCC%3DCC%3DC3%29C%28%3DO%29N2%29C%3DC1+&action=A) | [Non-substrate](http://lmmd.ecust.edu.cn/admetsar1/predict/?smiles=OC1%3DCC%3DC%28%5CC%3DN%5CNC2%3DN%5CC%28%3DC%2FC%3DC%2FC3%3DCC%3DCC%3DC3%29C%28%3DO%29N2%29C%3DC1+&action=A) | |
| CYP450 2D6 Substrate | [Non-substrate](http://lmmd.ecust.edu.cn/admetsar1/predict/?smiles=OC1%3DCC%3DC%28%5CC%3DN%5CNC2%3DN%5CC%28%3DC%2FC%3DC%2FC3%3DCC%3DCC%3DC3%29C%28%3DO%29N2%29C%3DC1+&action=A) | [Non-substrate](http://lmmd.ecust.edu.cn/admetsar1/predict/?smiles=OC1%3DCC%3DC%28%5CC%3DN%5CNC2%3DN%5CC%28%3DC%2FC%3DC%2FC3%3DCC%3DCC%3DC3%29C%28%3DO%29N2%29C%3DC1+&action=A) | | [Non-substrate](http://lmmd.ecust.edu.cn/admetsar1/predict/?smiles=OC1%3DCC%3DC%28%5CC%3DN%5CNC2%3DN%5CC%28%3DC%2FC%3DC%2FC3%3DCC%3DCC%3DC3%29C%28%3DO%29N2%29C%3DC1+&action=A) | [Non-substrate](http://lmmd.ecust.edu.cn/admetsar1/predict/?smiles=OC1%3DCC%3DC%28%5CC%3DN%5CNC2%3DN%5CC%28%3DC%2FC%3DC%2FC3%3DCC%3DCC%3DC3%29C%28%3DO%29N2%29C%3DC1+&action=A) | |
| CYP450 3A4 Substrate | [Substrate](http://lmmd.ecust.edu.cn/admetsar1/predict/?smiles=OC1%3DCC%3DC%28%5CC%3DN%5CNC2%3DN%5CC%28%3DC%2FC%3DC%2FC3%3DCC%3DCC%3DC3%29C%28%3DO%29N2%29C%3DC1+&action=A) | [Substrate](http://lmmd.ecust.edu.cn/admetsar1/predict/?smiles=OC1%3DCC%3DC%28%5CC%3DN%5CNC2%3DN%5CC%28%3DC%2FC%3DC%2FC3%3DCC%3DCC%3DC3%29C%28%3DO%29N2%29C%3DC1+&action=A) | | [Substrate](http://lmmd.ecust.edu.cn/admetsar1/predict/?smiles=OC1%3DCC%3DC%28%5CC%3DN%5CNC2%3DN%5CC%28%3DC%2FC%3DC%2FC3%3DCC%3DCC%3DC3%29C%28%3DO%29N2%29C%3DC1+&action=A) | [Substrate](http://lmmd.ecust.edu.cn/admetsar1/predict/?smiles=OC1%3DCC%3DC%28%5CC%3DN%5CNC2%3DN%5CC%28%3DC%2FC%3DC%2FC3%3DCC%3DCC%3DC3%29C%28%3DO%29N2%29C%3DC1+&action=A) | |
| CYP450 1A2 Inhibitor | [Non-inhibitor](http://lmmd.ecust.edu.cn/admetsar1/predict/?smiles=OC1%3DCC%3DC%28%5CC%3DN%5CNC2%3DN%5CC%28%3DC%2FC%3DC%2FC3%3DCC%3DCC%3DC3%29C%28%3DO%29N2%29C%3DC1+&action=A) | [Non-inhibitor](http://lmmd.ecust.edu.cn/admetsar1/predict/?smiles=OC1%3DCC%3DC%28%5CC%3DN%5CNC2%3DN%5CC%28%3DC%2FC%3DC%2FC3%3DCC%3DCC%3DC3%29C%28%3DO%29N2%29C%3DC1+&action=A) | | [Non-inhibitor](http://lmmd.ecust.edu.cn/admetsar1/predict/?smiles=OC1%3DCC%3DC%28%5CC%3DN%5CNC2%3DN%5CC%28%3DC%2FC%3DC%2FC3%3DCC%3DCC%3DC3%29C%28%3DO%29N2%29C%3DC1+&action=A) | [Non-inhibitor](http://lmmd.ecust.edu.cn/admetsar1/predict/?smiles=OC1%3DCC%3DC%28%5CC%3DN%5CNC2%3DN%5CC%28%3DC%2FC%3DC%2FC3%3DCC%3DCC%3DC3%29C%28%3DO%29N2%29C%3DC1+&action=A) | |
| CYP450 2C9 Inhibitor | [Non-inhibitor](http://lmmd.ecust.edu.cn/admetsar1/predict/?smiles=OC1%3DCC%3DC%28%5CC%3DN%5CNC2%3DN%5CC%28%3DC%2FC%3DC%2FC3%3DCC%3DCC%3DC3%29C%28%3DO%29N2%29C%3DC1+&action=A) | [Non-inhibitor](http://lmmd.ecust.edu.cn/admetsar1/predict/?smiles=OC1%3DCC%3DC%28%5CC%3DN%5CNC2%3DN%5CC%28%3DC%2FC%3DC%2FC3%3DCC%3DCC%3DC3%29C%28%3DO%29N2%29C%3DC1+&action=A) | | [Non-inhibitor](http://lmmd.ecust.edu.cn/admetsar1/predict/?smiles=OC1%3DCC%3DC%28%5CC%3DN%5CNC2%3DN%5CC%28%3DC%2FC%3DC%2FC3%3DCC%3DCC%3DC3%29C%28%3DO%29N2%29C%3DC1+&action=A) | [Non-inhibitor](http://lmmd.ecust.edu.cn/admetsar1/predict/?smiles=OC1%3DCC%3DC%28%5CC%3DN%5CNC2%3DN%5CC%28%3DC%2FC%3DC%2FC3%3DCC%3DCC%3DC3%29C%28%3DO%29N2%29C%3DC1+&action=A) | |
| CYP450 2D6 Inhibitor | [Non-inhibitor](http://lmmd.ecust.edu.cn/admetsar1/predict/?smiles=OC1%3DCC%3DC%28%5CC%3DN%5CNC2%3DN%5CC%28%3DC%2FC%3DC%2FC3%3DCC%3DCC%3DC3%29C%28%3DO%29N2%29C%3DC1+&action=A) | [Non-inhibitor](http://lmmd.ecust.edu.cn/admetsar1/predict/?smiles=OC1%3DCC%3DC%28%5CC%3DN%5CNC2%3DN%5CC%28%3DC%2FC%3DC%2FC3%3DCC%3DCC%3DC3%29C%28%3DO%29N2%29C%3DC1+&action=A) | | [Non-inhibitor](http://lmmd.ecust.edu.cn/admetsar1/predict/?smiles=OC1%3DCC%3DC%28%5CC%3DN%5CNC2%3DN%5CC%28%3DC%2FC%3DC%2FC3%3DCC%3DCC%3DC3%29C%28%3DO%29N2%29C%3DC1+&action=A) | [Non-inhibitor](http://lmmd.ecust.edu.cn/admetsar1/predict/?smiles=OC1%3DCC%3DC%28%5CC%3DN%5CNC2%3DN%5CC%28%3DC%2FC%3DC%2FC3%3DCC%3DCC%3DC3%29C%28%3DO%29N2%29C%3DC1+&action=A) | |
| CYP450 2C19 Inhibitor | [Non-inhibitor](http://lmmd.ecust.edu.cn/admetsar1/predict/?smiles=OC1%3DCC%3DC%28%5CC%3DN%5CNC2%3DN%5CC%28%3DC%2FC%3DC%2FC3%3DCC%3DCC%3DC3%29C%28%3DO%29N2%29C%3DC1+&action=A) | [Non-inhibitor](http://lmmd.ecust.edu.cn/admetsar1/predict/?smiles=OC1%3DCC%3DC%28%5CC%3DN%5CNC2%3DN%5CC%28%3DC%2FC%3DC%2FC3%3DCC%3DCC%3DC3%29C%28%3DO%29N2%29C%3DC1+&action=A) | | [Non-inhibitor](http://lmmd.ecust.edu.cn/admetsar1/predict/?smiles=OC1%3DCC%3DC%28%5CC%3DN%5CNC2%3DN%5CC%28%3DC%2FC%3DC%2FC3%3DCC%3DCC%3DC3%29C%28%3DO%29N2%29C%3DC1+&action=A) | [Non-inhibitor](http://lmmd.ecust.edu.cn/admetsar1/predict/?smiles=OC1%3DCC%3DC%28%5CC%3DN%5CNC2%3DN%5CC%28%3DC%2FC%3DC%2FC3%3DCC%3DCC%3DC3%29C%28%3DO%29N2%29C%3DC1+&action=A) | |
| CYP450 3A4 Inhibitor | [Non-inhibitor](http://lmmd.ecust.edu.cn/admetsar1/predict/?smiles=OC1%3DCC%3DC%28%5CC%3DN%5CNC2%3DN%5CC%28%3DC%2FC%3DC%2FC3%3DCC%3DCC%3DC3%29C%28%3DO%29N2%29C%3DC1+&action=A) | [Non-inhibitor](http://lmmd.ecust.edu.cn/admetsar1/predict/?smiles=OC1%3DCC%3DC%28%5CC%3DN%5CNC2%3DN%5CC%28%3DC%2FC%3DC%2FC3%3DCC%3DCC%3DC3%29C%28%3DO%29N2%29C%3DC1+&action=A) | | [Non-inhibitor](http://lmmd.ecust.edu.cn/admetsar1/predict/?smiles=OC1%3DCC%3DC%28%5CC%3DN%5CNC2%3DN%5CC%28%3DC%2FC%3DC%2FC3%3DCC%3DCC%3DC3%29C%28%3DO%29N2%29C%3DC1+&action=A) | [Non-inhibitor](http://lmmd.ecust.edu.cn/admetsar1/predict/?smiles=OC1%3DCC%3DC%28%5CC%3DN%5CNC2%3DN%5CC%28%3DC%2FC%3DC%2FC3%3DCC%3DCC%3DC3%29C%28%3DO%29N2%29C%3DC1+&action=A) | |
| **Excretion &Toxicity** | | | | | | |
| **hERG Inhibition** T_hERG_I | [Weak inhibitor](http://lmmd.ecust.edu.cn/admetsar1/predict/?smiles=BrC1%3DCC%3DC%28C%3DC1%29C1%3DCSC%28%3DN1%29N1N%3DC%28CC1C1%3DCC%3DCS1%29C1%3DCC%3DCS1+&action=A) | [Weak inhibitor](http://lmmd.ecust.edu.cn/admetsar1/predict/?smiles=BrC1%3DCC%3DC%28C%3DC1%29C1%3DCSC%28%3DN1%29N1N%3DC%28CC1C1%3DCC%3DCS1%29C1%3DCC%3DCS1+&action=A) | | [Weak inhibitor](http://lmmd.ecust.edu.cn/admetsar1/predict/?smiles=BrC1%3DCC%3DC%28C%3DC1%29C1%3DCSC%28%3DN1%29N1N%3DC%28CC1C1%3DCC%3DCS1%29C1%3DCC%3DCS1+&action=A) | [Weak inhibitor](http://lmmd.ecust.edu.cn/admetsar1/predict/?smiles=BrC1%3DCC%3DC%28C%3DC1%29C1%3DCSC%28%3DN1%29N1N%3DC%28CC1C1%3DCC%3DCS1%29C1%3DCC%3DCS1+&action=A) | |
| T_hERG_II | [Non-inhibitor](http://lmmd.ecust.edu.cn/admetsar1/predict/?smiles=BrC1%3DCC%3DC%28C%3DC1%29C1%3DCSC%28%3DN1%29N1N%3DC%28CC1C1%3DCC%3DCS1%29C1%3DCC%3DCS1+&action=A) | [Non-inhibitor](http://lmmd.ecust.edu.cn/admetsar1/predict/?smiles=BrC1%3DCC%3DC%28C%3DC1%29C1%3DCSC%28%3DN1%29N1N%3DC%28CC1C1%3DCC%3DCS1%29C1%3DCC%3DCS1+&action=A) | | [Non-inhibitor](http://lmmd.ecust.edu.cn/admetsar1/predict/?smiles=BrC1%3DCC%3DC%28C%3DC1%29C1%3DCSC%28%3DN1%29N1N%3DC%28CC1C1%3DCC%3DCS1%29C1%3DCC%3DCS1+&action=A) | [Non-inhibitor](http://lmmd.ecust.edu.cn/admetsar1/predict/?smiles=BrC1%3DCC%3DC%28C%3DC1%29C1%3DCSC%28%3DN1%29N1N%3DC%28CC1C1%3DCC%3DCS1%29C1%3DCC%3DCS1+&action=A) | |
| **AMES Toxicity** | [AMES toxic](http://lmmd.ecust.edu.cn/admetsar1/predict/?smiles=BrC1%3DCC%3DC%28C%3DC1%29C1%3DCSC%28%3DN1%29N1N%3DC%28CC1C1%3DCC%3DCS1%29C1%3DCC%3DCS1+&action=A) | [AMES toxic](http://lmmd.ecust.edu.cn/admetsar1/predict/?smiles=BrC1%3DCC%3DC%28C%3DC1%29C1%3DCSC%28%3DN1%29N1N%3DC%28CC1C1%3DCC%3DCS1%29C1%3DCC%3DCS1+&action=A) | | [AMES toxic](http://lmmd.ecust.edu.cn/admetsar1/predict/?smiles=BrC1%3DCC%3DC%28C%3DC1%29C1%3DCSC%28%3DN1%29N1N%3DC%28CC1C1%3DCC%3DCS1%29C1%3DCC%3DCS1+&action=A) | [AMES toxic](http://lmmd.ecust.edu.cn/admetsar1/predict/?smiles=BrC1%3DCC%3DC%28C%3DC1%29C1%3DCSC%28%3DN1%29N1N%3DC%28CC1C1%3DCC%3DCS1%29C1%3DCC%3DCS1+&action=A) | |
| **Carcinogens** | [Non-carcinogens](http://lmmd.ecust.edu.cn/admetsar1/predict/?smiles=BrC1%3DCC%3DC%28C%3DC1%29C1%3DCSC%28%3DN1%29N1N%3DC%28CC1C1%3DCC%3DCS1%29C1%3DCC%3DCS1+&action=A) | [Non-carcinogens](http://lmmd.ecust.edu.cn/admetsar1/predict/?smiles=BrC1%3DCC%3DC%28C%3DC1%29C1%3DCSC%28%3DN1%29N1N%3DC%28CC1C1%3DCC%3DCS1%29C1%3DCC%3DCS1+&action=A) | | [Non-carcinogens](http://lmmd.ecust.edu.cn/admetsar1/predict/?smiles=BrC1%3DCC%3DC%28C%3DC1%29C1%3DCSC%28%3DN1%29N1N%3DC%28CC1C1%3DCC%3DCS1%29C1%3DCC%3DCS1+&action=A) | [Non-carcinogens](http://lmmd.ecust.edu.cn/admetsar1/predict/?smiles=BrC1%3DCC%3DC%28C%3DC1%29C1%3DCSC%28%3DN1%29N1N%3DC%28CC1C1%3DCC%3DCS1%29C1%3DCC%3DCS1+&action=A) | |
| **Acute Oral Toxicity (AO)** | III | III | | III | III | |
| **Carcinogenicity (Three-class)** | non-required | non-required | | non-required | non-required | |
| **Biodegradation** | [Not ready biodegradable](http://lmmd.ecust.edu.cn/admetsar1/predict/?smiles=BrC1%3DCC%3DC%28C%3DC1%29C1%3DCSC%28%3DN1%29N1N%3DC%28CC1C1%3DCC%3DCS1%29C1%3DCC%3DCS1+&action=A) | [Not ready biodegradable](http://lmmd.ecust.edu.cn/admetsar1/predict/?smiles=BrC1%3DCC%3DC%28C%3DC1%29C1%3DCSC%28%3DN1%29N1N%3DC%28CC1C1%3DCC%3DCS1%29C1%3DCC%3DCS1+&action=A) | | [Not ready biodegradable](http://lmmd.ecust.edu.cn/admetsar1/predict/?smiles=BrC1%3DCC%3DC%28C%3DC1%29C1%3DCSC%28%3DN1%29N1N%3DC%28CC1C1%3DCC%3DCS1%29C1%3DCC%3DCS1+&action=A) | [Not ready biodegradable](http://lmmd.ecust.edu.cn/admetsar1/predict/?smiles=BrC1%3DCC%3DC%28C%3DC1%29C1%3DCSC%28%3DN1%29N1N%3DC%28CC1C1%3DCC%3DCS1%29C1%3DCC%3DCS1+&action=A) | |
